# Supplementary material for: Clinical pattern of checkpoint inhibitor-induced liver injury in a multicentre cohort
Source: JHEP Rep. 2023 Mar 7;5(6):100719. doi: 10.1016/j.jhepr.2023.100719 (PMC10149360; doi:10.1016/j.jhepr.2023.100719)
Supplement: Multimedia component 1 [file mmc1.pdf]

Journal of Hepatology, Volume ■

Supplemental information

Clinical pattern of checkpoint inhibitor-induced liver injury in a multi-centre cohort

Lina Hountondji, Christophe Ferreira De Matos, Fanny Lebossé, Xavier Quantin, Candice Lesage, Pascale Palassin, Valérian Rivet, Stéphanie Faure, Georges-Philippe Pageaux, Éric Assenat, Laurent Alric, Amel Zahaf, Dominique Larrey, Philine Witkowski Durand Viel, Benjamin Riviere, Selves Janick, Stéphane Dalle, Alexandre Thibault Jacques Maria, Thibaut Comont, and Lucy Meunier

Clinical pattern of checkpoint inhibitor-induced liver injury in a multicenter cohort

Lina Hountondji, Christophe Ferreira De Matos, Fanny Lebossé, Xavier Quantin, Candice Lesage, Pascale Palassin, Valérian Rivet, Stéphanie Faure, Georges-Philippe Pageaux, Éric Assenat, Laurent Alric, Amel Zahaf, Dominique Larrey, Philine Witkowski Durand Viel, Benjamin Riviere, Selves Janick, Stéphane Dalle, Alexandre Thibault Jacques Maria, Thibaut Comont[#], Lucy Meunier[#]

Table of contents:

Table S1: Clinico-biological comparison between cholestatic and hepatocellular patterns

Table S2: Clinico-biological features between immune-related sclerosing cholangitis and CHILI without macroscopic bile duct injury

Table S3: Comparison between treated and non-treated CHILI

Table S4: Comparison between steroids including treatment and other conditions

Table S5: Comparison between biopsied and non-biopsied patients

Table S6: Comparison between patients with and without rechallenge

Table S1: Clinico-biological comparison between cholestatic and hepatocellular patterns

	Cholestatic hepatitis n= 43 (36.8 %)	Hepatocellular hepatitis n= 45 (38.5 %)	*p value*
**Age (years), mean (SD)**	68	60	**0.004**
**Sex, n (%)**			0.133
Female	17 (19.3)	25 (28.4)	
Male	26 (29.5)	20 (22.7)	
**Cancer, n (%)**			0.091
Lung	15 (17)	10 (11.4)	
Melanoma	14 (15.9)	23 (26.1)	
Renal and urothelial	7 (8)	10 (11.4)	
Other cancer	7 (8)	2 (2.3)	
**Checkpoint inhibitor, n (%)**			**< 0.001**
Anti-PD1/PDL1	35 (39.8)	16 (18.2)	
Anti-CTLA4/Anti-LAG3	1 (1.1)	2 (2.3)	
Combotherapy with anti-CTLA4	7 (8)	27 (30.7)	
**Cycles of ICI infusion, mean (SD)**	4.9 (4.4)	3.5 (2.4)	0.64
**Time until onset (days), mean (SD)**	182.4 (262.5)	191.6 (372.1)	0.894
**RUCAM**			0.111
Possible (3-5)	0	4 (4.5)	
Probable (6-8)	28 (31.8)	24 (27.3)	
Highly probable (≥ 9)	15 (17)	17 (19.3)	
**Laboratory liver tests, mean/xULN (SD)**			
ALT	193.8 (151.9)	792.3 (1048.3)	**<0.001**
AST	166.4 (154.9)	535.3 (906.9)	**0.01**
GGT	670.7 (532.3)	202.1 (176.4)	**< 0.001**
ALP	804.4 (1687.2)	177.6 (124.9)	**0.015**
Total bilirubin	32.4 (45.7)	19.8 (24.2)	0.109
Jaundice (total bilirubin > N)	12 (10.3)	10 (11.4)	0.538
**Autoantibodies**			
ANA only	7 (8)	5 (5.7)	0.480
ASMA	0	5 (5.7)	**0.023**
**Bile duct injury, n (%)**	8 (9.1)	0	**0.002**
**Liver biopsy, n (%)**	20 (22.7)	23 (26.1)	0.666
**Histology, n (%)**			
Biliary injury	16 (39)	6 (14.6)	**< 0.001**
Granuloma	4 (10.3)	4 (10.3)	0.997
Endothelitis	0	3 (10.3)	**0.042**
Fibrin ring granuloma	1 (2.6)	0	0.299
**Other irAEs, n (%)**			
Extra hepatic irAE	20 (22.7)	13 (14.8)	0.088
Gastrointestinal	6 (6.8)	4 (4.5)	0.454
Cutaneous	4 (4.5)	7 (8)	0.375
Endocrine	4 (4.5)	3 (3.4)	0.648
Other irAE*	10 (11.4)	3 (3.4)	**0.028**
Multiple irAEs	7 (8)	6 (6.8)	0.171
**Hepatitis treatment, n (%)**			
Steroids only	15 (17)	31 (35.2)	**0.005**
UDCA only	5 (5.7)	0	**0.005**
Steroids + UDCA	18 (20.5)	10 (11.4)	**0.005**
Steroid including regimen	33 (37.5)	41 (46.6)	0.065
No treatment	5 (5.7)	4 (4.5)	0.672
Second line treatment	7 (8)	9 (10.2)	0.651
**Days until resolution to grade 1, mean (SD)**	69.5 (50)	59 (49.4)	0.488

Table S2: Clinico-biological features between immune-related sclerosing cholangitis and CHILI without macroscopic bile duct injury

	Bile duct stenosis n= 8 (6.8 %)	No bile duct stenosis 109 (93.2 %)	*p value*
**Age (years), mean (SD)**	65.3 (8.9)	63.2 (14.2)	0.686
**Sex, n (%)**			0.197
Female	2 (1.7)	52 (42.5)	
Male	6 (5.2)	55 (47.8)	
**Cancer, n (%)**			0.273
Lung	3 (2.6)	30 (26.1)	
Melanoma	1 (0.9)	47 (40.9)	
Renal and urothelial	2 (1.7)	20 (17.4)	
Other cancer	2 (1.7)	10 (8.7)	
**Checkpoint inhibitor, n (%)**			0.254
Anti-PD1/PDL1	7 (6.1)	62 (53.9)	
Anti-CTLA4/Anti-LAG3	0	4 (3.5)	
Combotherapy with anti-CTLA4	1 (0.9)	41 (35.7)	
**Cycles of ICI infusion, mean (SD)**	6.9 (3.4)	4.4 (4.6)	0.137
**Time until onset (days), mean (SD)**	236 (197.7)	173.6 (296.4)	0.560
**RUCAM**			0.754
Possible (3-5)	0	7 (6.1)	
Probable (6-8)	5 (4.3)	64 (55.7)	
Highly probable (≥ 9)	3 (2.6)	36 (31.3)	
**Laboratory liver tests, mean/xULN (SD)**			
ALT	180.5 (107.8)	463.1 (741.8)	0.286
AST	127.6 (136.6)	327.2 (620.2)	0.367
GGT	1027.4 (821.7)	353.3 (330.5)	**< 0.001**
ALP	1818.9 (3776.8)	319.7 (369.2)	**< 0.001**
Total bilirubin	18.2 (23.9)	24.2 (34.4)	0.628
Jaundice (total bilirubin > N)	2 (1.7)	26 (22.6)	0.964
**Autoantibodies**			
ANA only	0	16 (13.9)	0.238
ASMA	0	6 (5.3)	0.489
**Liver biopsy, n (%)**	6 (5.2)	43 (37.4)	0.055
**Histology, n (%)**			
Biliary injury	5 (10.6)	21 (44.7)	0.139
Granuloma	0	9 (20.4)	0.409
Endothelitis	0	4 (11.4)	0.334
Fibrin ring granuloma	0	1 (2.3)	0.688
**Other irAEs, n (%)**			
Extra hepatic irAE	5 (4.3)	42 (36.5)	0.197
Gastrointestinal	2 (1.7)	10 (8.7)	0.162
Cutaneous	0	13 (11.3)	0.295
Endocrine	2 (1.7)	9 (7.8)	0.124
Other irAE*	2 (1.7)	17 (14.8)	0.503
Multiple irAEs	1 (0.9)	19 (16.5)	**0.002**
**Hepatitis treatment, n (%)**			
Steroids only	1 (0.9)	60 (52.2)	**0.008**
UDCA only	1 (0.9)	6 (5.2)	**0.008**
Steroids + UDCA	6 (5.2)	25 (21.7)	**0.008**
Steroid including regimen	7 (6.1)	85 (73.9)	0.582
No treatment	0	16 (13.9)	0.238
Second line treatment	3 (2.6)	15 (13)	0.078
**Days until resolution to grade 1, mean (SD)**	124 (26.4)	50.5 (45)	**< 0.001**

Table S3: Comparison between treated and non-treated CHILI

	Treatment n= 100 (85.5 %)	No treatment n= 17 (14.5 %)	*p value*
**Age (years), mean (SD)**	63.6 (13.9)	61.2 (13.8)	0.512
**Sex, n (%)**			0.544
Female	45 (38.5)	9 (7.7)	
Male	55 (47)	8 (6.8)	
**Cancer, n (%)**			0.565
Lung	29 (24.8)	5 (4.3)	
Melanoma	44 (37.6)	5 (4.3)	
Renal and urothelial	18 (15.4)	4 (3.4)	
Other cancer	9 (7.7)	3 (2.6)	
**Checkpoint inhibitor, n (%)**			0.508
Anti-PD1/PDL1	58 (49.6)	12 (10.3)	
Anti-CTLA4/Anti-LAG3	4 (3.4)	0	
Combotherapy with anti-CTLA4	38 (32.5)	5 (4.3)	
**Pattern, n (%)**			0.066
Cholestatic	38 (32.5)	5 (4.3)	
Mixed	21 (17.9)	8 (6.8)	
Cytolytic	41 (35)	4 (3.4)	
**Cycles of ICI infusion, mean (SD)**	4.4 (4.7)	5.4 (4)	0.405
**Time until onset (days), mean (SD)**	181.1 (307)	144.6 (135.7)	0.632
**RUCAM**			0.456
Possible (3-5)	7 (6)	0	
Probable (6-8)	61 (52.1)	10 (8.5)	
Highly probable (≥ 9)	32 (27.4)	7 (6)	
**Laboratory liver tests, mean/xULN (SD)**			
ALT	469.4 (763.9)	284.4 (220.6)	0.325
AST	332.2 (640.5)	203.1 (161.6)	0.412
GGT	419.4 (435.1)	336.2 (288.4)	0.450
ALP	454.7 (1145.4)	245.5 (179.3)	0.455
Total bilirubin	25 (35.5)	15.6 (14.2)	0.299
Jaundice (total bilirubin > N)	26 (22.2)	2 (1.7)	0.203
**Autoantibodies**			
ANA only	14 (12)	2 (1.7)	0.804
ASMA	6 (5.2)	0	0.295
**Bile duct injury, n (%)**	8 (7)	0	0.238
**Liver biopsy, n (%)**	45 (38.5)	4 (3.4)	0.097
**Histology, n (%)**			
Biliary injury	23 (48.9)	3 (6.4)	0.408
Granuloma	8 (18.1)	1 (2.3)	0.148
Endothelitis	4 (11.4)	0	0.515
Fibrin ring granuloma	1 (2.3)	0	0.784
**Other irAEs, n (%)**			
Extra hepatic irAE	41 (35)	7 (6)	0.989
Gastrointestinal	12 (10.3)	0	0.132
Cutaneous	13 (11.1)	0	0.115
Endocrine	10 (8.5)	1 (0.9)	0.591
Other irAE*	18 (15.4)	2 (1.7)	0.528
Multiple irAEs	19 (16.2)	1 (0.9)	0.121
**Days until resolution to grade 1, mean (SD)**	63.8 (49.9)	23.2 (14.6)	**0.007**
**Cancer status (RECIST 1.1), n (%)**			0.278
Progressive disease	23 (20)	7 (6.1)	
Stable disease	13 (11.3)	4 (3.5)	
Partial response	34 (29.6)	4 (3.5)	
Complete response	20 (17.4)	1 (0.9)	
**CHILI recurrence, n (%)**	9 (19.6)	3 (6.5)	0.153

Table S4: Comparison between steroids including treatment and other conditions*

	Steroids including regimen n= 93 (79.5 %)	Other treatments n= 24 (20.5 %)	*p value*
**Age (years), mean (SD)**	63.4 (14.1)	62.7 (13)	0.827
**Sex, n (%)**			0.672
Female	42 (35.9)	12 (10.3)	
Male	51 (43.6)	12 (10.3)	
**Cancer, n (%)**			0.193
Lung	26 (22.2)	8 (6.8)	
Melanoma	42 (35.9)	7 (6)	
Renal and urothelial	18 (15.4)	4 (3.4)	
Other cancer	7 (6)	5 (4.3)	
**Checkpoint inhibitor, n (%)**			0.082
Anti-PD1/PDL1	51 (43.6)	19 (16.2)	
Anti-CTLA4/Anti-LAG3	4 (3.4)	0	
Combotherapy with anti-CTLA4	38 (32.5)	5 (4.3)	
**Pattern, n (%)**			**0.025**
Cholestatic	33 (28.2)	10 (8.5)	
Mixed	19 (16.2)	10 (8.5)	
Hepatocellular	41 (35)	4 (3.4)	
**Cycles of ICI infusion, mean (SD)**	4.5 (4.8)	4.7 (3.6)	0.799
**Time until onset (days), mean (SD)**	169.7 (294.9)	199.2 (266.4)	0.658
**RUCAM**			0.292
Possible (3-5)	7 (6)	0	
Probable (6-8)	57 (48.7)	14 (12)	
Highly probable (≥ 9)	29 (24.8)	10 (8.5)	
**Laboratory liver tests, mean/xULN (SD)**			
ALT	488.6 (787.7)	263.9 (208.9)	0.170
AST	339.1 (661.1)	213.9 (182.6)	0.361
GGT	393.9 (410.8)	458.9 (444.2)	0.506
ALP	441.4 (1167.9)	356 (484.8)	0.726
Total bilirubin	24.9 (36.1)	18.7 (19.6)	0.429
Jaundice (total bilirubin > N)	24 (20.5)	4 (3.4)	0.349
**Autoantibodies**			
ANA only	13 (11.1)	3 (2.6)	0.851
ASMA	6 (5.2)	0	0.196
**Bile duct injury, n (%)**	7 (6.1)	1 (0.9)	0.582
**Liver biopsy, n (%)**	42 (35.9)	7 (6)	0.157
**Histology, n (%)**			
Biliary injury	21 (44.7)	5 (10.6)	0.353
Granuloma	8 (18.1)	1 (2.3)	0.378
Endothelitis	4 (11.4)	0	0.334
Fibrin ring granuloma	1 (2.3)	0	0.688
**Other irAEs, n (%)**			
Extra hepatic irAE	38 (32.5)	10 (8.5)	0.943
Gastrointestinal	12 (10.3)	0	0.063
Cutaneous	13 (11.1)	0	0.052
Endocrine	9 (7.7)	2 (1.7)	0.841
Other irAE*	16 (13.7)	4 (3.4)	0.950
Multiple irAEs	19 (16.2)	1 (0.9)	0.080
**Second-line immunosuppressant, n (%)**	18 (15.4)	0	**0.019**
**Days until resolution to grade 1, mean (SD)**	23.2 (14.6)	63.8 (49.9)	**0.007**
**Cancer status (RECIST 1.1), n (%)**			0.222
Progressive disease	21 (18.3)	9 (7.8)	
Stable disease	12 (10.4)	5 (4.3)	
Partial response	31 (27)	7 (6.1)	
Complete response	20 (17.4)	1 (0.9)	

**CHILI recurrence, n (%)**	8 (17.4)	4 (8.7)	0.257

*UDCA only or no treatment

Table S5: Comparison between biopsied and non-biopsied patients

	Biopsy n= 49 (41.9 %)	No biopsy n= 68 (58.1 %)	*p value*
**Age (years), mean (SD)**	62.8 (15)	63.6 (13)	0.758
**Sex, n (%)**			0.370
Female	25 (21.4)	29 (24.8)	
Male	24 (20.5	39 (33.3)	
**Cancer, n (%)**			0.109
Lung	13 (11.1)	21 (17.9)	
Melanoma	19 (16.2)	30 (25.6)	
Renal and urothelial	14 (12)	8 (6.8)	
Other cancer	3 (2.6)	9 (7.7)	
**Checkpoint inhibitor, n (%)**			0.243
Anti-PD1/PDL1	26 (22.2)	44 (37.6)	
Anti-CTLA4/Anti-LAG3	3 (2.6)	1 (0.9)	
Combotherapy with anti-CTLA4	20 (17.1)	23 (19.7)	
**Cycles of ICI infusion, mean (SD)**	4.4 (4.1)	4.5 (4.9)	0.892
**Time until onset (days), mean (SD)**	184.8 (233.6)	169.2 (323.7)	0.774
**RUCAM**			0.427
Possible (3-5)	2 (1.7)	5 (4.3)	
Probable (6-8)	33 (28.2)	38 (32.5)	
Highly probable (≥ 9)	14 (12)	25 (21.4)	
**Laboratory liver tests, mean (SD)**			
ALT	608.1 (1010.2)	323.2 (340)	**0.033**
AST	415.02 (857.3)	240.2 (276.7)	0.118
GGT	526.2 (516.1)	317.1 (295.7)	0.007
ALP	577.2 (1572.3)	314.2 (393.9)	0.188
Total bilirubin	31.6 (45)	17.8 (19.6)	**0.028**
Jaundice (total bilirubin > N)	16 (13.7)	12 (10.3)	0.061
**Autoantibodies**			
ANA only	7 (6)	9 (7.7)	0.870
ASMA	4 (3.5)	2 (1.7)	0.221
**Bile duct injury, n (%)**	6 (5.2)	2 (1.7)	0.055
**Other irAEs, n (%)**			
Extra hepatic irAE	15 (12.8)	33 (28.2)	0.052
Gastrointestinal	3 (2.6)	9 (7.7)	0.211
Cutaneous	4 (3.4)	9 (7.7)	0.389
Endocrine	4 (3.4)	7 (6)	0.697
Other irAE	5 (4.3)	15 (12.8)	0.093
Multiple irAEs	6 (5.1)	14 (12)	0.244
**Hepatitis treatment, n (%)**			
Corticoids only	3 (18.8)	40 (34.2)	0.19
UDCA only	3 (2.6)	4 (3.4)	0.19
Corticoids + UDCA	20 (17.1)	11 (9.4)	0.19
Corticoids including regimen	42 (35.9)	51 (43.6)	0.157
No treatment	4 (3.4)	13 (11.1)	0.097
Second line treatment	16 (13.7)	2 (1.7)	**< 0.001**
**Days until resolution to grade 1, mean (SD)**	75.2 (54.5)	42.4 (37.7)	0.006

Table S6: Comparison between patients with and without rechallenge

	Rechallenge n= 51 (43.6%)	No rechallenge n= 66 (56.4%)	*p value*
**Age (years), mean (SD)**	61.3 (15.3)	64.7 (12.5)	0.182
**Sex, n (%)**			0.863
Female	24 (20.5)	30 (25.6)	
Male	27 (23.1)	36 (30.8)	
**Pre-existing liver disease, n (%)**			
Cirrhosis	1 (0.9)	4 (3.4)	0.277
**Cancer, n (%)**			0.644
Lung	15 (12.8)	19 (16.2)	
Melanoma	23 (19.7)	26 (22.2)	
Renal and urothelial	7 (6)	15 (12.8)	
Other cancer	6 (5.1)	6 (5.1)	
**Checkpoint inhibitor, n (%)**			0.197
Anti-PD-1/PDL-1	31 (26.5)	39 (33.3)	
Anti-CTLA-4/Anti-LAG-3	0	4 (3.4)	
Combi-therapy with anti-CTLA-4	20 (17.1)	23 (19.7)	
**Cycles of ICI infusion, mean (SD)**	4.4 (5)	4.6 (4.3)	0.894
**Time until onset (days), mean (SD)**	137.5 (198.2)	205.4 (340.8)	0.208
**Autoantibodies**			
ANA only	9 (7.7)	7 (6)	0.272
ASMA	2 (1.7)	4 (3.5)	0.577
**Pattern, n (%)**			0.312
Cholestatic	16 (13.7)	27 (23.1)	
Mixed	16 (13.7)	13 (11.1)	
Hepatocellular	19 (16.2)	26 (22.2)	
**Severity (CTCAE), n (%)**			0.006
Grade 1	1 (0.9)	3 (2.6)	
Grade 2	13 (11.1)	4 (3.4)	
Grade 3	32 (27.4)	41 (35)	
Grade 4	5 (4.3)	18 (15.4)	
**Jaundice (total bilirubin > N)**	9 (7.7)	19 (16.2)	0.161
**Bile duct injury, n (%)**	3 (2.6)	5 (4.3)	0.686
**Liver biopsy, n (%)**	13 (11.1)	36 (30.8)	0.002
**Histology, n (%)**			
Biliary injury	6 (12.8)	20 (42.6)	0.435
Granuloma	5 (11.4)	4 (9)	0.093
Endothelitis	1 (2.9)	3 (8.6)	0.593
Fibrin ring granuloma	1 (2.3)	0	0.118
**Other irAEs, n (%)**			
Extra hepatic irAE	19 (16.2)	29 (24.8)	0.466
Gastrointestinal	4 (3.4)	8 (6.8)	0.449
Cutaneous	8 (6.8)	5 (4.3)	0.166
Endocrine	6 (5.1)	5 (4.3)	0.441
Other irAE*	7 (6)	13 (11.1)	0.395
Multiple irAEs (>2)	10 (8.5)	10 (8.5)	0.397
**Hepatitis treatment, n (%)**			
Corticoids including regimen	39 (33.3)	54 (46.2)	0.477
No treatment	8 (6.8)	9 (7.7)	0.755
Other immunosuppressant	5 (4.3)	13 (11.1)	0.141
**Days until resolution to grade 1, mean (SD)**	47.5 (36.9)	65.5 (56.7)	0.136
**Cancer status (RECIST 1.1), n (%)**			0.533
Progressive disease	14 (12.2)	16 (13.9)	
Stable disease	9 (7.8)	8 (7)	
Partial response	17 (14.8)	21 (18.3)	
Complete response	6 (5.2)	15 (13)	
Unmesurable	5 (4.3)	4 (3.5)	

Table S7 : Histology

	Biopsied patients n= 49 (%)
**Inflammation and necrosis**	
Portal inflammation	35 (71.4)
Portal necrosis	9 (18.4)
Lobular inflammation	41 (83.7)
Lobular necrosis	24 (49)
Centrilobular injury	14 (28.6)
Panlobular injury	17 (34.7)
Interface hepatitis	5 (10.2)
Portal endothelitis	3 (6.1)
Centro venular endothelitis	1 (2)
**Cellular infiltrate**	46 (93.9)
Lymphocytes	34 (69.4)
Neutrophils	21 (42.9)
Eosinophils	25 (51)
Histiocytes	16 (32.7)
Macrophages	20 (40.8)
Plama cells	13 (26.5)
Granuloma	10 (20.4)
Fibrin ring granuloma	1 (2)
Biliary injury	26 (53)
Bilirubinostasis	4 (8.1)
Ductopenia	2 (4.1)
Ductular reaction	10 (20.4)
Ductular dystrophia	13 (26.5)
Inflammatory cholangitis	15 (30.6)
Lymphocytic cholangitis	9 (18.4)
Neutrophilic cholangitis	2 (4.1)
Non-suppurative destructive cholangitis	3 (6.1)
Granulomatous cholangitis	2 (4.1)
Obstructive fibrous pericholangiolitis	0
**Fibrosis**	16 (32.7)
F0	31 (63.3)
Portal fibrosis (F1)	11 (22.4)
Centrolobular fibrosis (F1)	5 (10.2)
Fibrosis with septa (F2)	0
Fibrosis with numerous septa (F3)	0
Cirrhosis (F4)	1 (2)
