# Supplementary material for: Clinical pattern of checkpoint inhibitor-induced liver injury in a multicentre cohort
Source: JHEP Rep. 2023 Mar 7;5(6):100719. doi: 10.1016/j.jhepr.2023.100719 (PMC10149360; doi:10.1016/j.jhepr.2023.100719)
Supplement: Multimedia component 4 [file mmc4.pdf]

Clinical pattern of checkpoint inhibitor-induced liver injury in a multicentre cohort

Lina Hountondji,[1,†] **Christophe Ferreira De Matos**,[2,†] Fanny Lebossé,[3] Xavier Quantin,[4] Candice Lesage,[5] Pascale Palassin,[6] Valérian Rivet,[2] Stéphanie Faure,[1] Georges-Philippe Pageaux,[1] Éric Assenat,[7] Laurent Alric,[8] Amel Zahhaf,[9] Dominique Larrey,[9] Philine Witkowski Durand Viel,[10] Benjamin Riviere,[11] Selves Janick,[12] Stéphane Dalle,[13] Alexandre Thibault Jacques Maria,[14] Thibaut Comont,[2,†] Lucy Meunier[9,†,*]

[1]Department of Liver Transplantation, Saint Eloi Hospital, Montpellier University Hospital, Montpellier, France; [2]Department of Internal Medicine, IUCT-Oncopole, Toulouse University Hospital, Toulouse, France; [3]Department of Hepatology, Croix Rousse Hospital, Lyon Liver Institute, Hospices Civils of Lyon, Lyon, France; [4]Department of Medical Oncology, Montpellier Cancer Institute, Montpellier University Hospital, Montpellier, France; [5]Department of Dermatology, Saint Eloi Hospital, Montpellier University Hospital, Montpellier, France; [6]Department of Medical Pharmacology and Toxicology, Lapeyronie Hospital, Montpellier University Hospital, Montpellier, France; [7]Department of Oncology, Saint Eloi Hospital, Montpellier University Hospital, Montpellier, France; [8]Department of Internal Medicine and Digestive Diseases, Purpan Hospital, Toulouse University Hospital, Toulouse, France; [9]Department of Liver Transplantation, Saint Eloi Hospital, Montpellier University Hospital, REFHEPS, Montpellier, France; [10]Internal Medicine, Beziers Hospital, France; [11]Department of Pathology, Montpellier University Hospital, University of Montpellier, Montpellier, France; [12]Department of Pathology, Oncopole, Toulouse, France; [13]Department of Dermatology, Lyon Sud Hospital, Lyon Cancer Institute, Hospices Civils of Lyon, Lyon, France; [14]Internal Medicine & Immuno-Oncology (MedI2O), Institute for Regenerative Medicine and Biotherapy (IRMB), Saint Eloi Hospital, Montpellier University Hospital, Montpellier, France

JHEP Reports **2023.** https://doi.org/10.1016/j.jhepr.2023.100719

Background & Aims: Immune checkpoint inhibitors (ICIs) have changed the landscape of cancer therapy. Liver toxicity occurs in up to 25% of patients treated with ICIs. The aim of our study was to describe the different clinical patterns of ICI-induced hepatitis and to assess their outcome.
Methods: We conducted a retrospective observational study of patients with checkpoint inhibitor-induced liver injury (CHILI) discussed in multidisciplinary meetings between December 2018 and March 2022 in three French centres specialised in ICI toxicity management (Montpellier, Toulouse, Lyon). The hepatitis clinical pattern was analysed by the ratio of serum alanine aminotransferase (ALT) and alkaline phosphatase (ALP) (R value = (ALT/ULN)/(ALP/ULN)) for characterisation as cholestatic ($R \leq 2$), hepatocellular ($R \geq 5$), or mixed ($2 < R < 5$).
Results: We included 117 patients with CHILI. The clinical pattern was hepatocellular in 38.5%, cholestatic in 36.8%, and mixed in 24.8% of patients. High-grade hepatitis severity (grade ≥3 according to the Common Terminology Criteria for Adverse Events system) was significantly associated with the hepatocellular hepatitis ($p < 0.05$). No cases of severe acute hepatitis were reported. Liver biopsy was performed in 41.9% of patients: granulomatous lesions, endothelitis, or lymphocytic cholangitis were described. Biliary stenosis occurred in eight patients (6.8%) and was significantly more frequent in the cholestatic clinical pattern ($p < 0.001$). Steroids alone were mainly administered to patients with a hepatocellular clinical pattern (26.5%), and ursodeoxycholic acid was more frequently used in the cholestatic pattern (19.7%) than in the hepatocellular or mixed clinical pattern ($p < 0.001$). Seventeen patients improved without any treatment. Among the 51 patients (43.6%) rechallenged with ICIs, 12 (23.5%) developed CHILI recurrence.
Conclusions: This large cohort indicates the different clinical patterns of ICI-induced liver injury and highlights that the cholestatic and hepatocellular patterns are the most frequent with different outcomes.
Impact and Implications: ICIs can induce hepatitis. In this retrospective series, we report 117 cases of ICI-induced hepatitis, mostly grades 3 and 4. We find a similar distribution of the different patterns of hepatitis. ICI could be resumed without systematic recurrence of hepatitis.
© 2023 The Authors. Published by Elsevier B.V. on behalf of European Association for the Study of the Liver (EASL). This is an open access article under the CC BY license (http://creativecommons.org/licenses/by/4.0/).

Keywords: Immune checkpoint inhibitors; Immune-related adverse events; Drug-induced liver injury; Cancer; Hepatitis; Cholangitis.
Received 10 January 2023; received in revised form 10 February 2023; accepted 16 February 2023; available online 7 March 2023
[†] These authors contributed equally.
* Corresponding author. Address: Department of Liver Transplantation, Saint Eloi Hospital, Montpellier University Hospital, REFHEPS, 80 avenue Augustin Fliche, 34090 Montpellier, France. Tel: +33 4 67 33 02 24, Fax: +33 4 67 33 69 42
E-mail address: Lucy.meunier@chu-montpellier.fr (L. Meunier).

ELSEVIER
EASL The Home of Hepatology

Introduction

Immune checkpoint inhibitors (ICIs) are an evolving class of antitumour immunotherapy drugs that have become a cornerstone for cancer treatment in the past decade by revolutionising the prognosis of many advanced solid and haematological neoplasia.[1,2] Indeed, ICIs are in full expansion and have been approved for many cancers since 2010.[3–5] Indications for treatment with ICIs are still currently growing.[6,7]

Current ICIs are monoclonal antibodies targeting inhibitory receptors on the surface of T cells or tumour cells.[8] These inhibitory receptors include PD-1 (programmed cell death-1), its ligand PDL-1 (programmed cell death ligand-1), and CTLA-4 (cytotoxic T-lymphocyte-associated protein-4). Recently, there is a new ICI targeting the inhibitory receptor LAG-3 (lymphocyte-activation gene 3).[9] Unlike conventional chemotherapies that have a direct cytotoxic effect on tumour cells, ICIs aim to reverse the tumour cell-driven and immune checkpoint-mediated inhibition of antitumour cytotoxic T-cell activity. However, via restoring antitumour immunity, ICIs can induce multisystem immune-related adverse events (irAEs) that can affect up to 90% of treated patients according to the immune checkpoint targeted and the drug combination.[10]

Checkpoint inhibitor-induced liver injury (CHILI) develops in up to 25% of patients treated with ICIs and primarily among patients under combination therapy involving an anti-CTLA-4.[11] In contrast, the incidence of severe hepatitis requiring ICI discontinuation has been reported at below 5% (grade ≥3 CHILI according to the Common Terminology Criteria for Adverse Events [CTCAE] severity classification).[12] Consensus guidelines recommend suspending immunotherapy and initiating low-dose corticosteroids (0.5–1 mg/kg/day) for CTCAE grade 2 or high-dose corticosteroids for CTCAE grade ≥3.[13,14] However, these international recommendations were based on small patient inclusions with heterogeneous hepatitis clinical pattern. In addition, some case series studies have shown that 37.5–50% of patients with severe acute hepatitis can improve without treatment with corticosteroids.[12,15] In the case of corticosteroid-resistant hepatitis, many agents have been studied and appear to be effective: mycophenolate mofetil (MMF), azathioprine, ciclosporin, tacrolimus, and anti-thymocyte globulin. Nonetheless, data remain scarce regarding the benefit–risk balance in the context of cancer.

Immune-mediated sclerosing cholangitis has been more recently recognised as a secondary sclerosing cholangitis induced by ICIs.[16,17] The response of patients with ICI-induced sclerosing cholangitis to steroids has been found as low as 11.5%.[17] Moreover, a case series study has shown that patients with severe steroid-resistant cholestatic hepatitis, induced by anti-PD-1, may benefit from ursodeoxycholic acid (UDCA) treatment.[18] Finally, despite case series studies reporting a hepatitis recurrence rate of between 25.8 and 35% after ICI rechallenge,[19] to date, there is still a lack of data for the official recommendation of ruling out ICI rechallenge following CHILI.

Overall, there is great heterogeneity in the clinical presentation, evolution, and outcomes of CHILI (with or without treatment with steroids) as well as a lack of up-to-date recommendations for ICI rechallenge in daily practice. Therefore, we collected a large number of cases having developed CHILI to evaluate the clinical characteristics and evolution of CHILI, as well as response to treatment and risk of recurrence after rechallenge according to the clinical pattern of drug-induced liver injury (DILI): cholestatic, hepatocellular, or mixed.

Patients and methods

Patients and data collection

We conducted a retrospective study of patients with CHILI discussed during multidisciplinary meetings between December 2018 and March 2022 in three French university centres specialised in ICI toxicity (Montpellier, Toulouse, and Lyon). Inclusion criteria were (i) patients with previous normal liver tests (defined as normal transaminase and bilirubin levels); (ii) ICI-treated patients; and (iii) patients having developed a clinical presentation of CHILI after other causes of hepatitis had been ruled out. Patients with underlying liver disease but with normal baseline liver tests were included for study. CHILI-related data were collected at diagnosis; after Weeks 1, 2 and 4; and then weekly until recovery from hepatitis. Data regarding cancer, treatment of CHILI, and ICI rechallenge were also collected.

Characterisation of the hepatitis

Diagnosis of CHILI was defined according to CTCAEv5 criteria as patients enrolled presented with normal baseline liver tests. CHILI was defined by elevations in alanine aminotransferase (ALT) >3-fold the upper limit of normal (ULN) (CTCAE grade 1), ALT >3- to 5-fold the ULN (CTCAE grade 2), ALT >5- to 20-fold the ULN (CTCAE grade 3), or ALT >20-fold the ULN (CTCAE grade 4) in participants with normal previous liver tests (defined as normal transaminase and bilirubin levels).

The Drug-Induced Liver Injury Network (DILIN) score was also used for grading liver injury severity as mild, moderate, moderate to severe, severe, or fatal according to the presence of jaundice, international normalised ratio >1.5, hospitalisation, liver or other organ failure, liver transplant, or death.[20]

All patients were referred to the liver unit, and an extensive workup was carried out to rule out other causes of liver enzyme abnormalities, including viral hepatitis, autoimmune disease, cancer progression, vascular complications, or other potential treatments causing DILI. Liver imaging (ultrasound, computed tomography, or magnetic resonance imaging) was systematically performed. Liver biopsy was carried out at the discretion of the referring physician. The Roussel Uclaf Causality Assessment Method (RUCAM) was used to assess the causality of CHILI diagnosis after ICI treatment; a RUCAM score ≥5 was retained for a positive diagnosis of CHILI.[21,22] The hepatitis pattern was analysed by the serum ALT and ALP ratio (R value = (ALT/ULN)/(ALP/ULN)) and categorised as cholestatic (R ≤2), hepatocellular (R ≥5), or mixed (2 <R <5).

Statistical analysis

Descriptive statistics are presented as medians (ranges) for quantitative variables and frequencies (percentages) for qualitative variables. The Wilcoxon rank-sum test was applied for comparing the distribution of continuous variables, and the Chi-squared test (or Fisher's exact test when appropriate) was used to test for association between categorical variables. Data were statistically analysed using two-way ANOVA. A p value <0.05 was considered statistically significant, and all statistical tests were two-sided. All statistical analyses were performed using the SPSS

software version 28.0 (IBM Corporation, Armonk, NY, USA). The study was approved by the local research ethics committee (IRB ID 202100908).

Results

Patient baseline characteristics

Among the patients discussed during multidisciplinary meetings in Montpellier (n = 479), Toulouse (n = 329), and Lyon (n = 250) between December 2018 and March 2022 for ICI toxicity, 145 patients had abnormal liver tests. After exclusion of 28 patients (four for HEV infection), 117 patients with CHILI were included for study (Fig. 1).

The characteristics of the cohort are summarised in Table 1. The median age was 63 (23–89) years, with a sex ratio of 1.2 (63 males, 53.8%). Twenty-two patients had pre-existing liver disease.

ICIs were mostly used for treating melanoma (n = 49, 41.9%), non-small cell lung cancer and squamous cell lung carcinoma (n = 33, 28%), and renal cell carcinoma (n = 16, 13.7%). Cancer was localised (stage I–II) in 19.6% (n = 23) and advanced (stage III–IV) in 48% (n = 56) of patients. Most patients received PD-1 inhibitors (n = 104, 88.9%), either alone (n = 62, 53%) or with a CTLA-4 inhibitor (n = 42, 35.6%). The RUCAM scores were as expected in majority probable (6–8) and highly probable (≥9) (n = 110, 94%). CHILI was associated with non-hepatic irAEs in 48 patients (41%), including cutaneous (13/48, 27%), gastrointestinal (12/48, 25%), and endocrine (11/48, 22.9%) disorders.

General characteristics of CHILI

The CHILI pattern was cholestatic in 36.8% (n = 43), hepatocellular in 38.5% (n = 45), and mixed in 24.8% (n = 29) of patients. There was no difference in sex ratio, age, cancer type, mean number of ICI infusion cycles, and mean time to onset of CHILI between these three groups (Table 2). Regarding severity, 96 patients (82.1%) were CTCAE grade ≥3, as shown in Fig. 2: grade 3 (n = 73, 62%) and grade 4 (n = 23, 19.7%). The DILIN severity score was mild in 72 patients (61.5%) and moderate in 45 patients (38.5%). There were no patients with acute liver failure. Severity was significantly associated with clinical pattern; CTCAE grade 4 was more frequently associated with a hepatocellular pattern (n = 18, 40%; p <0.05). Anti-CTLA-4 inhibitor-containing regimen was also significantly associated with severity; 13 patients under such regimen developed CTCAE grade 4 hepatitis (13/45 *vs.* 9/71 for non-CTLA-4-treated patients, p <0.001). Moreover, the clinical pattern was significantly associated with the ICI used; a hepatocellular pattern was more frequent under anti-CTLA-4 inhibitor-containing

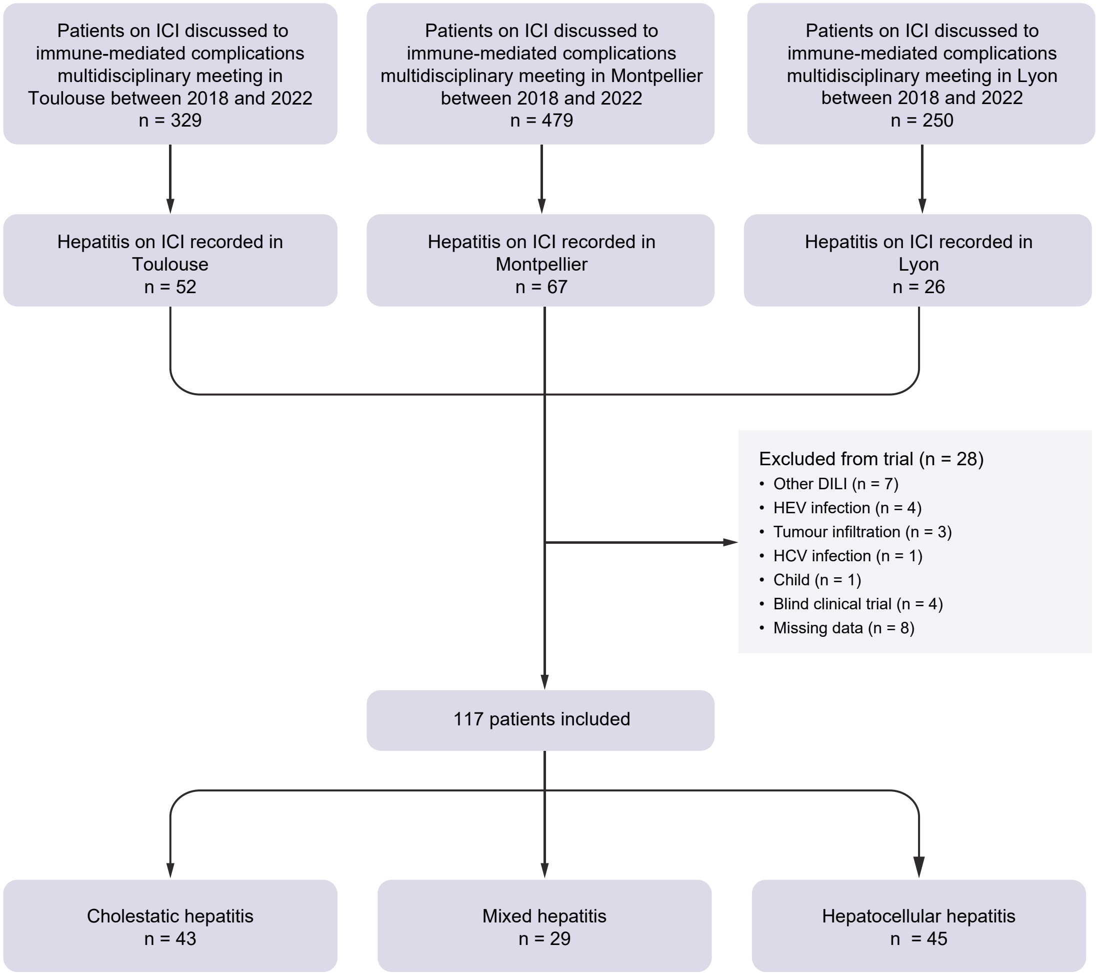

Fig. 1. Flow chart of the study. DILI, drug-induced liver injury; ICI, immune checkpoint inhibitor.

Table 1. Characteristics of patients with immune checkpoint-induced liver injury.

	Value, N = 117
Age at diagnosis (years), median (range)	63 (23–89)
Sex, n (%)	
Female	54 (46.2)
Male	63 (53.8)
Medical history, n (%)	
Chronic alcohol consumption	9 (11)
IgG-4 mesenteric panniculitis	1 (0.9)
Diabetes	18 (15.5)
Liver transplant	1 (0.9)
Pre-existing liver disease, n (%)	
Liver metastasis	14 (11.9)
Cirrhosis	5 (4.3)
Chronic viral hepatitis	3 (2.6)
Cancer, n (%)	
Melanoma	49 (41.9)
Lung	32 (27.3)
Renal	16 13.7)
Urothelial	6 (5.1)
Cutaneous and oral squamous cell carcinoma	7 (5.9)
Gastrointestinal	3 (2.6)
Hepatocellular carcinoma	2 (1.7)
Haematological malignancies	1 (0.9)
Pancreatic adenocarcinoma	1 (0.9)
Cancer stage, n (%)	
Stage I–II	23 (19.6)
Stage III	13 (11.1)
Stage IV	43 (36.8)
Unknown	38 (32.5)
Baseline hepatic biochemistries (×ULN), median (range)	
ALT	1 (1–5)
AST	1 (1–3)
ALP	1 (1–3.6)
GGT	1 (1–4)
Total bilirubin	1 (1–1.6)
Checkpoint inhibitor, n (%)	
Anti-PD-1	62 (53)
Anti-PDL-1	8 (6.8)
Anti-CTLA-4	4 (3.4)
Anti-PD-1 + anti-CTLA-4	42 (35.9)
Anti-PD-1 + anti-LAG-3	1 (0.9)
RUCAM, n (%)	
Possible (4–5)	7 (6)
Probable (6–8)	71 (60.7)
Highly probable (≥9)	39 (33.3)
Severity (CTCAE), n (%)	
Grade 1	4 (3.4)
Grade 2	17 (14.5)
Grade 3	73 (62.4)
Grade 4	23 (19.7)
Grade 5	0
DILIN severity score, n (%)	
Mild	72 (61.5)
Moderate	45 (38.5)
Severe	0

Continuous values are provided as median and IQR.
ALP, alkaline phosphatase; ALT, alanine aminotransferase; AST, aspartate aminotransferase; CTCAE, Common Terminology Criteria for Adverse Events; CTLA-4, cytotoxic T-lymphocyte-associated protein-4; DILIN, Drug-Induced Liver Injury Network; GGT, gamma-glutamyl-transferase; LAG-3, lymphocyte-activation gene 3; PD-1, programmed cell death-1; PDL-1, programmed cell death ligand-1; RUCAM, Roussel Uclaf Causality Assessment Method; ULN, upper limit normal.

regimen (n = 27, 31%; *p* <0.001), and a cholestatic pattern was more frequent with anti-PD-1/PDL-1 inhibitors (n = 35, 30%; *p* <0.001).

Liver biopsy was performed in 49 patients (41.9%): 20 in the cholestatic pattern (41%), 23 in the hepatocellular pattern (47%), and six in the mixed pattern (12%). Granulomatous lesions were similar among the three groups (*p* = 0.697). When comparing cholestatic and hepatocellular patterns (Table S1), endothelitis was significantly more frequent in the hepatocellular pattern (*p* = 0.042). Endothelitis was observed in three patients under anti-CTLA-4/anti-LAG-3-containing regimen, and a specific histological lesion of fibrin ring granuloma was only observed in one patient under anti-CTLA-4 immunotherapy. Over half of the biopsies performed in the cholestatic group showed biliary injury with lymphocytic cholangitis, non-suppurative destructive or granulomatous cholangitis, and/or ductal dystrophy (Fig. 3). Furthermore, computed tomography scan or magnetic resonance imaging detected bile duct injury in eight patients (7%), with bile duct injury being significantly associated with the cholestatic pattern (cholestatic, n = 8; hepatocellular and mixed, n= 0; *p* <0.001). Bile duct injury was radiologically defined as the development of bile duct stenosis without obstruction after the initiation of ICI therapy (Fig. 3). Bile duct injury tended to be more associated with anti PD-1/PDL-1 regimen (n = 7, 88%; *p* = 0.254).

Treatment of CHILI

CHILI spontaneously resolved without any treatment in 17 patients (14.5%): five patients (29.4%) had CTCAE grade 2 hepatitis, 11 patients (64.7%) grade 3 hepatitis, and one patient (5.9%) grade 4 hepatitis. Other patients were treated with steroids alone (n = 62, 53%), with steroids and UDCA (n = 31, 26.5%), or with UDCA alone (n = 7, 6%). Steroids alone were mainly administered to patients developing a hepatocellular pattern (n = 31, 26.5%; *p* <0.001) and UDCA-containing regimen were more frequently used in patients developing a cholestatic pattern (n = 23, 19.7%; *p* <0.001) (Table 2). However, 18 patients (15.3%) received second-line immunosuppressive treatment (Table 3): 17 patients received MMF (94%) and one patient received rituximab. Immunosuppressive therapy was indicated, according to the guidelines, in case of resistance or dependence of the hepatitis to corticosteroids. CHILI severity was CTCAE grade 3 in 11 patients (61%) and grade 4 in seven patients (39%). The CHILI pattern did not affect the likelihood of receiving second-line treatment (*p* = 0.306).

Evolution of CHILI

The mean time until resolution of the hepatitis was similar among the three patterns (*p* = 0.356). This delay was significantly longer among patients with bile duct injury (17.7 ± 3.8 weeks; *p* <0.001) than among those without bile duct injury (7.2 ± 6.4 weeks). The type of cancer (*p* = 0.026), the presence of macroscopic bile duct injury (*p* = 0.021), and the need for second-line immunosuppressive therapy (*p* = 0.030) were significantly associated with the resolution of hepatitis (Fig. 4).

The mean time until hepatitis resolution was significantly longer among patients who underwent second-line treatment (14.1 *vs.* 7.4 weeks; *p* = 0.035) and significantly shorter among untreated patients (23.2 *vs.* 63.8 days; *p* = 0.007) than among patients who underwent first-line treatment only.

All patients recovered after second-line treatment, and no patients developed liver failure. After a median follow-up of 48.6 (1.6–228.6) weeks, no CHILI-related deaths were reported, but 25 patients (22%) died because of cancer progression.

Rechallenge with ICI and recurrence of CHILI

Fifty-one patients (43.6%) underwent ICI rechallenge, including 37 patients with CTCAE grade ≥3 hepatitis (Table 4). The same ICI

Table 2. Clinico-biological and histological characteristics, and treatment in the three groups of the study.

	Cholestatic hepatitis n = 43 (36.8%)	Mixed hepatitis n = 29 (24.8%)	Hepatocellular hepatitis n = 45 (38.5%)	*p* value
Age (years), mean (SD)	67.8 (13.7)	63.2 (9.9)	58.8 (14.9)	0.106
Sex, n (%)				0.269
Female	17 (14.5)	12 (10.3)	25 (21.4)	
Male	26 (22.2)	17 (14.5)	20 (17.1)	
Cancer, n (%)				0.365
Lung	15 (12.8)	9 (7.7)	10 (8.5)	
Melanoma	14 (12)	12 (10.3)	23 (19.7)	
Renal and urothelial	7 (6)	5 (4.3)	10 (8.5)	
Other cancer	7 (6)	3 (2.6)	2 (1.7)	
Checkpoint inhibitor, n (%)				<0.001
Anti-PD-1/PDL-1	35 (29.9)	19 (16.2)	16 (13.7)	
Anti-CTLA-4/Anti-LAG-3	1 (0.9)	1 (0.9)	2 (1.7)	
Combination therapy with anti-CTLA-4	7 (6)	9 (7.7)	27 (23.1)	
Cycles of ICI infusion, mean (SD)	4.9 (4.4)	5.4 (6.7)	3.5 (2.4)	0.170
Time until onset (days), mean (SD)	182.4 (262.4)	141.3 (148.3)	191.6 (372.1)	0.754
RUCAM				0.213
Possible (3–5)	0	3 (2.6)	4 (3.4)	
Probable (6–8)	28 (23.9)	19 (16.2)	24 (20.5)	
Highly probable (≥9)	15 (12.8)	7 (6)	17 (14.5)	
Laboratory liver tests, mean (SD)				
ALT	193.8 (151.9)	268.6 (156.3)	792.3 (1048.3)	<0.001
AST	166.4 (154.9)	187.2 (129.4)	535.3 (906.9)	0.005
GGT	670.7 (532.3)	350.1 (276.8)	202.1 (176.4)	<0.001
ALP	804.4 (1687.2)	243.4 (177.8)	177.6 (124.9)	0.011
Total bilirubin	32.4 (45.7)	16.6 (20.1)	19.8 (24.2)	0.94
Jaundice (total bilirubin >N)	12 (10.3)	6 (5.1)	10 (8.5)	0.736
Autoantibodies				
ANA only	7 (6)	4 (3.4)	5 (4.3)	0.780
ASMA	0	1 (0.9)	5 (4.3)	0.053
Bile duct injury, n (%)	8 (7)	0	0	<0.001
Liver biopsy, n (%)	20 (17.1)	6 (5.1)	23 (19.7)	0.026
Histology, n (%)				
Biliary injury	16 (34)	4 (8.5)	6 (12.8)	0.003
Granuloma	4 (9.1)	1 (2.3)	4 (9.1)	0.697
Endothelitis	0	1 (2.9)	3 (8.6)	0.137
Fibrin ring granuloma	1 (2.3)	0	0	0.51
Other irAEs, n (%)				
Extrahepatic irAE	20 (17.1)	15 (12.8)	13 (11.1)	0.098
Gastrointestinal	6 (5.1)	2 (1.7)	4 (3.4)	0.581
Cutaneous	4 (3.4)	2 (1.7)	7 (6)	0.457
Endocrine	4 (3.4)	4 (3.4)	3 (2.6)	0.591
Other irAE*	10 (8.5)	7 (6)	3 (2.6)	0.06
Multiple irAEs (>2)	7 (6)	7 (6)	6 (5.1)	0.294
Hepatitis treatment, n (%)				
Steroids only	15 (12.8)	16 (13.7)	31 (26.5)	0.001
UDCA only	5 (4.3)	2 (1.7)	0	0.001
Steroids + UDCA	18 (15.4)	3 (2.6)	10 (8.5)	0.001
Steroid-including regimen	33 (28.2)	19 (16.2)	41 (35)	0.025
No treatment	5 (4.3)	8 (6.8)	4 (3.4)	0.066
Second-line treatment†	7 (6)	2 (1.7)	9 (7.7)	0.306
Days until steroids introduction, mean (SD)	18.7 (32.1)	7.3 (13.3)	5.6 (6.4)	0.002
Median (IQR)	8 (2–22)	2 (1–9)	3 (1–8)	
Days until UDCA introduction, mean (SD)	36 (30)	33.8 (22.1)	17.1 (12.1)	0.033
Median (IQR)	25 (19–56)	38.5 (27–45.25)	15 (7.25–27.5)	
Days until resolution to grade 1, mean (SD)	69.5 (50)	38.5 (40.4)	59 (49.4)	0.356

Data are expressed as mean (SD).
The Chi-square test was used for comparison between qualitative variables, and ANOVA and Student's *t* test were used for quantitative variables.
Levels of significance: p <0.05. ANA, N <1/160; ASMA, N <1/40.
ALP, alkaline phosphatase; ALT, alanine aminotransferase; ANA, antinuclear antibodies; ASMA, antismooth muscle antibodies; AST, aspartate aminotransferase; GGT, gamma-glutamyl-transferase; CTLA-4, cytotoxic T-lymphocyte-associated protein-4; ICI, immune checkpoint inhibitor; irAE, immune-related adverse event; LAG-3, lymphocyte-activation gene 3; PD-1, programmed cell death-1; PDL-1, programmed cell death ligand-1; RUCAM, Roussel Uclaf Causality Assessment Method; UDCA, ursodeoxycholic acid.
* Pneumological, cardiologic, rheumatologic, neurologic, haematologic, and nephrological.
† Mycophenolate mofetil, rituximab, or tacrolimus.

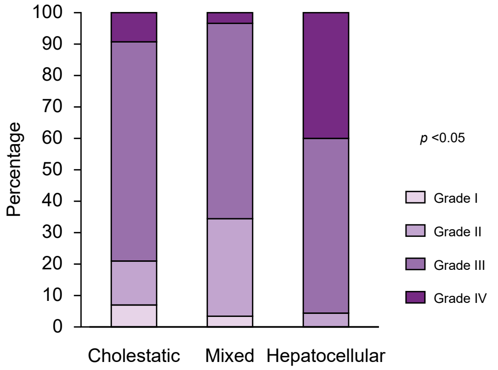

Fig. 2. Immune checkpoint-induced liver injury severity assessed by CTCAE severity score. The Chi-square test with Yate's correction was used. Levels of significance: p <0.05. CTCAE, Common Terminology Criteria for Adverse Events.

was reintroduced for 31 patients (31/51, 60.8%) and mainly by monotherapy (49/51, 96.1%). Two patients (CTCAE grades 2 and 3) were rechallenged with combination therapy (nivolumab/ ipilimumab) mainly owing to their young age and partial oncological response. At the time of rechallenge, treatment for CHILI was continued in 35 patients: low-dose steroid (5–15 mg of prednisone or budesonide) in 26 patients, steroid and UDCA in three patients, UDCA only in five patients, and MMF in one patient. Recurrence of CHILI occurred after rechallenge in 12 patients (12/51, 23.5%). Severity of CHILI recurrence was CTCAE grade 2 in four patients (4/12, 33.3%), grade 3 in five patients (5/ 12, 41.7%), and grade 4 in three patients (3/12, 25%). The former CHILI pattern did not affect the likelihood of CHILI recurrence (p = 0.651), and the CHILI recurrence rate was similar among patients rechallenged with anti-PD-1/PDL-1- or anti-CTLA-4-containing regimens. The rate of patients with significant (titres ≥1:160) antinuclear antibodies (ANA) (p = 0.581) and

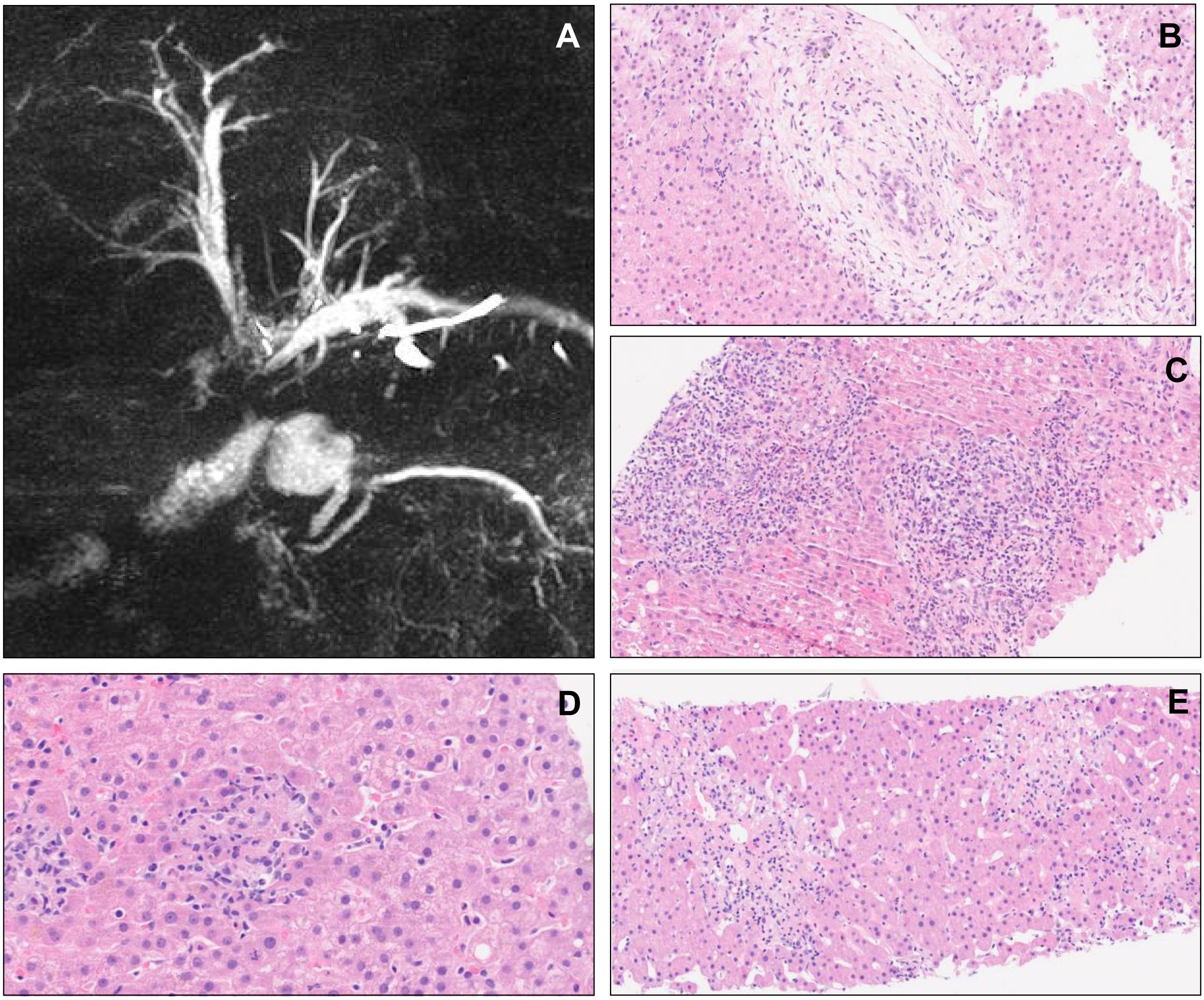

Fig. 3. Morphologic assessment and liver biopsies. (A) Magnetic resonance imaging showing sclerosing cholangitis. (B) Lymphocytic cholangitis (HES 20×). (C) Neutrophilic cholangitis (HES 20×). (D) Granulomatous hepatitis (HES 40×). (E) Pericentrolobular necrosis and endothelitis (HES 20×). HES, Hemalun-Eosine-Safran coloration.

Table 3. Main characteristics of patients with second-line immunosuppressant.

Patient	Sex, age (years); ancer, sites of metastases	Previous ICI exposure	ICI agents	Time to CHILI onset (days)	Other irAEs	CHILI phenotype, CTCAE grade	Serum autoantibody	Serum IgG (g/L)	Histology	First-line treatment for CHILI	Second-line treatment, time from CHILI (days)	Time until resolution to grade 1 (days)
1	M, 49; melanoma, lymph node	No	Combo ipilimumab + nivolumab	20	Vitiligo, thyroiditis	Cholestatic, grade 3	ANA 1/320, ASMA negative	6.84	Interface hepatitis, eosinophilia, fibrin ring granulomas	Steroids 1.5 mg/kg, UDCA 500 mg	Mycophenolate mofetil, 92	136
2	F, 54; melanoma, lymph node	No	Pembrolizumab	220	No	Cholestatic, grade 3	ANA negative, ASMA negative	4.33	Interface hepatitis, eosinophilia, mixed cholangitis*	Steroids 1 mg/kg, UDCA 750 mg	Mycophenolate mofetil, 198	138
3	M, 67; hypopharynx, lung	No	Nivolumab	95	Interstitial pneumonia	Cholestatic, grade 4	ANA negative, ASMA negative	6.20	Portal fibrosis (F1)	Steroids 2 mg/kg, UDCA 500 mg	Rituximab 1 g/2 weeks, 483	
4	F, 30; melanoma, lymph node and brain	Pembrolizumab	Combo ipilimumab + nivolumab	62	No	Hepatocellular, grade 3	ANA negative, ASMA 1/160	9.9	Panlobular necrotising hepatitis	Steroids 2 mg/kg	Mycophenolate mofetil, 113	154
5	F, 84; melanoma, lung	Pembrolizumab, + combo ipilimumab + nivolumab	Nivolumab	13	No	Hepatocellular, grade 3	ANA negative, ASMA negative	7.7	Lobular necrotising hepatitis, histiocytes	Steroids 2 mg/kg, UDCA 1,000 mg	Mycophenolate mofetil, 30	62
6	F, 60; melanoma, lung and liver	Pembrolizumab	Combo ipilimumab + nivolumab	11	Colitis, dermatitis	Cholestatic, grade 3	ANA negative, ASMA negative	n.a.	None	Steroids 1 mg/kg, UDCA 1,500 mg	Mycophenolate mofetil, 114	79
7	F, 40; melanoma, lung and liver	Nivolumab	Ipilimumab	37	Thyroiditis	Hepatocellular, grade 4	ANA negative, ASMA negative	n.a.	Panlobular necrotising hepatitis, histiocytes	Steroids 1 mg/kg	Mycophenolate mofetil, 30	180
8	F, 79; melanoma, lymph node and lung	Combo ipilimumab + nivolumab	Nivolumab	434	Vitiligo, thyroiditis	Hepatocellular, grade 3	ANA positive, ASMA 1/100	21	Centrolobular fibrosis (F1)	Steroids 2 mg/kg, UDCA 1,000 mg	Mycophenolate mofetil, 18	69
9	M, 54; renal cell carcinoma, lymph node and lung	No	Combo ipilimumab + nivolumab	54	No	Hepatocellular, grade 4	ANA positive, ASMA 1/100	10	Lobular necrotising hepatitis, histiocytes, cholangitis	Steroids 2 mg/kg, UDCA 1,000 mg	Mycophenolate mofetil, 52	90
10	F, 60; melanoma, liver and spleen	No	Combo ipilimumab + nivolumab	65	No	Hepatocellular, grade 4	ANA negative, ASMA negative	12.6	Plasmocytes, histiocytes	Steroids 4 mg/kg, UDCA 1,500 mg	Mycophenolate mofetil, 21	82
11	M, 55; renal cell carcinoma, adrenal	No	Combo ipilimumab + nivolumab	20	No	Hepatocellular, grade 4	ANA negative, ASMA negative	11.3	Panlobular necrotising hepatitis, plasmocytes, histiocytes	Steroids 1.25 mg/kg, UDCA 750 mg	Mycophenolate mofetil, 8	74
12	F, 63; lung cancer, bone	No	Pembrolizumab	33	No	Cholestatic, grade 3	ANA negative, ASMA negative	14.9	Cholangitis, plasmocytes, histiocytes	Steroids 2 mg/kg, UDCA 1,000 mg	Mycophenolate mofetil, 15	170
13	F, 77; lung cancer, adrenal	No	Pembrolizumab	93	No	Cholestatic, grade 4	ANA negative, ASMA negative	4.3	Cholangitis, ductopaenia	Steroids 2 mg/kg, UDCA 1,000 mg	Mycophenolate mofetil, 23	187
14	M, 53; lung cancer, lymph node and brain	No	Combo ipilimumab + nivolumab	62	No	Hepatocellular, grade 4	ANA negative, ASMA negative	10.7	None	Steroids 1 mg/kg	Mycophenolate mofetil, 9	120
15	M, 55; renal cell carcinoma, bone	Combo ipilimumab + nivolumab	Nivolumab	133	Colitis	Cholestatic, grade 3	ANA negative, ASMA negative	8	Cholangitis	Steroids 2 mg/kg, UDCA 1,000 mg	Mycophenolate mofetil, 147 (tacrolimus, abatacept)	549
16	M, 74; melanoma, lung, brain, and adrenal	No	Combo ipilimumab + nivolumab	48	Dermatitis, myocarditis	Hepatocellular, grade 3	ANA negative, ASMA negative	n.a.	Centrolobular necrotising hepatitis, plasmocytes, centrolobular fibrosis (F1)	Steroids 2 mg/kg, UDCA 1,000 mg	Mycophenolate mofetil, 18	54
17	M, 61, renal cell carcinoma, lung	Combo ipilimumab + nivolumab	Nivolumab	161	Hypophysitis, thyroiditis, myocarditis	Mixed, grade 3	ANA negative, ASMA negative	8	Lobular necrotising hepatitis, cholangitis, portal fibrosis (F1)	Steroids 2 mg/kg	Mycophenolate mofetil, 27	162
18	F, 49; melanoma, peritoneum	No	Combo ipilimumab + nivolumab	42	No	Mixed, grade 3	ANA negative, ASMA negative	n.a.	Centrolobular hepatitis, portal fibrosis (F1)	Steroids 2 mg/kg	Mycophenolate mofetil, 7	15

ANA, N <1/160; ASMA, N <1/40.
ANA, antinuclear antibodies; ASMA, antismooth muscle antibodies; CHILI, checkpoint inhibitor-induced liver injury; CTCAE, Common Terminology Criteria for Adverse Events; ICI, immune checkpoint inhibitor; irAE, immune-related adverse event; n.a., not available.
* Mixed cholangitis, lymphocytic cholangitis, and non-suppurative destructive cholangitis.

Table 4. Recurrence rate of CHILI.

	Recurrence n = 12 (23.5%)	No recurrence n = 39 (76.5%)	*p* value
**Baseline characteristics**			
Age (years), mean (SD)	63.8 (14.2)	60.3 (16.5)	0.523
Sex, n (%)			0.089
Female	8 (17.4)	13 (28.3)	
Male	4 (8.7)	21 (45.7)	
Pre-existing liver disease, n (%)			
Liver metastasis	0	5 (10.9)	0.159
Cirrhosis	0	1 (2.2)	0.548
Cancer, n (%)			0.574
Lung	4 (8.7)	9 (19.6)	
Melanoma	4 (8.7)	17 (37)	
Renal and urothelial	1 (2.2)	5 (10.9)	
Other cancer	2 (4.3)	3 (6.5)	
**Previous CHILI characteristics**			
Checkpoint inhibitor, n (%)			0.514
Anti-PD-1/PDL-1	8 (17.4)	19 (41.3)	
Combination therapy with anti-CTLA-4	4 (8.7)	15 (32.6)	
Cycles of ICI infusion, mean (SD)	3.9 (2.8)	4.3 (5.7)	0.827
Time until onset (days), mean (SD)	156.8 (179.3)	129.4 (218.7)	0.699
Autoantibodies			
ANA only	3 (6.5)	6 (13)	0.581
ASMA	0	2 (4.3)	0.390
Pattern, n (%)			0.651
Cholestatic	4 (8.7)	11 (23.9)	
Mixed	5 (10.9)	10 (21.7)	
Hepatocellular	3 (6.5)	13 (28.3)	
Severity (CTCAE), n (%)			0.830
Grade 1	0	1 (2.2)	
Grade 2	2 (4.3)	8 (17.4)	
Grade 3	9 (19.6)	21 (45.7)	
Grade 4	1 (2.2)	4 (8.7)	
Bile duct injury, n (%)	2 (4.3)	1 (2.2)	0.098
Liver biopsy, n (%)	4 (8.7)	9 (19.6)	0.650
Histology, n (%)			
Biliary injury	4 (30.8)	2 (15.4)	**0.009**
Granuloma	1 (7.7)	4 (30.8)	0.713
Endothelitis	1 (7.7)	0	0.118
Fibrin ring granuloma	0	1 (7.7)	0.488
Treatment, n (%)			
Corticoids only	6 (13)	20 (43.5)	0.553
UDCA only	1 (2.2)	3 (6.5)	0.553
Corticoids + UDCA	2 (4.3)	8 (17.4)	0.553
Corticoid-including regimen	8 (17.4)	28 (60.9)	0.257
No treatment	3 (6.5)	3 (6.5)	0.153
Other immunosuppressant	0	5 (10.9)	0.159
Initial dose of corticoids (mg/day), mean (SD)	666.7 (288.7)	805.6 (242.9)	0.429
**Characteristics at ICI rechallenge**			
Same checkpoint inhibitor, n (%)	9 (19.6)	18 (39.1)	0.182
Checkpoint inhibitor at rechallenge, n (%)			0.258
Anti-PD-1	10 (21.7)	33 (71.8)	
Anti-PDL-1	0	1 (2.2)	
Anti-PD-1 + anti-CTLA-4	1 (2.2)	1 (2.2)	
Simultaneous treatment, n (%)	7 (17.5)	28 (70)	0.316
Cancer status (RECIST 1.1), n (%)			0.570
Progressive disease	2 (4.3)	11 (23.9)	
Stable disease	3 (6.5)	3 (6.5)	
Partial response	5 (10.9)	12 (26.1)	
Complete response	1 (2.2)	5 (10.9)	
Other irAEs, n (%)	1 (8.3)	0	0.640

Data are expressed as mean (SD).
The Chi-square test was used for comparison between qualitative variables, and ANOVA and Student's *t* test were used for quantitative variables.
Levels of significance: p <0.05. ANA, N <1/160; ASMA, N <1/40.
ANA, antinuclear antibodies; ASMA, antismooth muscle antibodies; CHILI, checkpoint inhibitor-induced liver injury; CTCAE, Common Terminology Criteria for Adverse Events; CTLA-4, cytotoxic T-lymphocyte-associated protein-4; ICI, immune checkpoint inhibitor; irAE, immune-related adverse event; PD-1, programmed cell death-1; PDL-1, programmed cell death ligand-1; RECIST 1.1, Response Evaluation Criteria in Solid Tumours 1.1; UDCA, ursodeoxycholic acid.

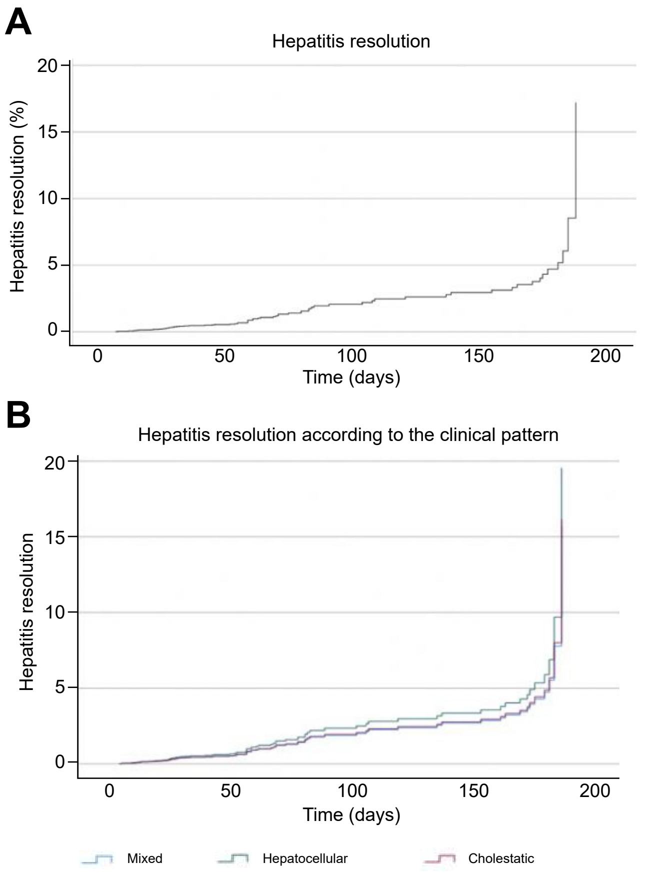

Fig. 4. Hepatitis resolution estimated using the Kaplan–Meyer method. (A) Overall hepatitis resolution. (B) Hepatitis resolution stratified by clinical pattern. Hepatitis resolution rates were estimated using the Cox test regression and Kaplan–Meier method in percentage.

antismooth muscle antibodies (ASMA) (p = 0.390) was similar between patients with and without recurrence. Previous biliary injury was significantly associated with the likelihood of recurrence (30.8%; p = 0.009). Among patients with recurrence of CHILI, seven patients (7/12, 58.3%) underwent ICI rechallenge with steroids only (4/7, 57.1%), UDCA only (1/7, 14.3%), or steroids with UDCA (2/7, 28.6%). ICI rechallenge containing steroids did not modify the risk of CHILI recurrence (p = 0.316). Overall, one patient (1/51, 2%) developed a different irAE after ICI rechallenge (immune-related haemolytic anaemia), and 19 patients had recurrence of their previous irAE (19/51, 37.3%) (Table S6).

Discussion

This retrospective observational study describes a cohort of 117 patients with CHILI, including 96 patients with CTCAE grade ≥3 hepatitis. Using the ratio R (ALT/ULN)/(ALP/ULN), as used in other forms of DILI, we found that CHILI develops with various clinical patterns: cholestatic, hepatocellular, and mixed.[23]

In this study, the cholestatic (36.8%) and hepatocellular (38.5%) patterns presented at approximately the same frequency. The hepatocellular pattern was significantly associated with hepatitis severity and anti-CTLA-4 inhibitor-containing regimen. In contrast, the cholestatic pattern was associated with microscopic biliary injury and anti-PD(L)-1 inhibitor regimen.

Among the cholestatic group, eight patients had macroscopic bile duct injury with image-detected dilatation or stenosis. These forms of secondary sclerosing cholangitis have already been reported in the literature.[24–26] In our study, all the patients with macroscopic biliary injury had the cholestatic pattern. These results support the idea that radiological assessment is relevant for evaluating macroscopic bile duct injury, especially in patients with cholestatic CHILI. All patients with bile duct injury had been treated with anti PD(L)-1-containing regimen alone, and not with anti-CTL-4 alone as reported by Takinami *et al.*[24] in their case series study. Previously, Stein *et al.*[27] evoked a link between PDL-1 blockade and cholangitis in mice. The authors showed that $CD8^+$ T-cell-derived IL-17 induced the expression of PDL-1 on antigen-presenting cholangiocytes and limited the expansion of self-reactive T cells in cholangitis. PDL-1 presented by antigen-presenting cells is well known to inhibit T-cell proliferation after binding to the PD-1 receptor, and cholangiocytes have been previously described to upregulate the expression of PDL-1 *in vitro*, protecting themselves by reducing $CD8^+$ T-cell cytotoxicity. Thus, it can be assumed that anti-PD(L)-1 can induce cholangitis by promoting $CD8^+$ T-cell cytotoxicity in cholangiocytes.

Interestingly, IgG-4-related disease, a cause of secondary sclerosing cholangitis, has also been reported in association with cancer and could be a novel paraneoplastic entity. Recently, a few cases of IgG-4-related disease related to pancreatitis, pleural involvement, and cholangitis have been reported.[28–30] In our patient cohort, only one patient with severe interstitial pneumonia and refractory cholangitis had histological features fulfilling the criteria of IgG-4-related disease. We thus decided to treat this patient with rituximab, and the outcome was complete resolution of both aforementioned irAEs. We think clinicians should be aware of this peculiar association and thus consider measuring serum IgG-4 levels and investigate IgG-4 positive staining in cases of ICI-induced cholangitis with lymphoplasmacytic infiltrates and biliary duct involvement.

Liver biopsy was performed in CTCAE grade ≥2 hepatitis in our study, as recommended, and at the discretion of the referring physicians in the university hospital centres. Liver biopsy analysis was available for 49 patients in our cohort. Endothelitis was significantly associated with the hepatocellular pattern, whereas granulomatous hepatitis developed in all patterns and was not exclusively associated with anti-CTLA-4-containing regimen, contrary to first reports in the literature.[12] There is yet no histological data regarding anti-LAG-3-induced liver injury; this ICI was only recently approved by the FDA.[9] However, our study suggests that endotheliitis could be associated with anti-LAG-3-induced liver injury. Biliary injury occurred in all patterns but was significantly more frequent in the cholestatic pattern with primary biliary cholangitis-like biliary injury, such as lymphocytic cholangitis and non-suppurative destructive or granulomatous cholangitis. These results are consistent with those from Cohen *et al.*,[31] finding an association between the cholestatic pattern and histologic biliary duct injury. This correlation between biological pattern and histology could therefore limit the need of performing routine liver biopsies in these patients. Instead, liver biopsy could be reserved for cases evoking differential diagnosis or cases with aggravation despite optimal treatment.

Another important finding from this study is the discrepancy between the grade of hepatitis according to the CTCAE system *vs.* the DILIN score. Although the majority of CHILIs were CTCAE grade ≥3 in our study, none of the patients evoked criteria of severe hepatitis, required transplantation, or died. The CTCAE

classification does not correlate with liver function and should not be used alone to assess the severity of hepatitis.

Regarding treatment in this study, 79.5% of patients were treated with steroid-containing regimen, and 14.5% of patients with CHILI spontaneously recovered without any treatment, even patients with high-grade hepatitis (10.3%). This evolution has already been reported by several teams among 38–50% of patients.[12,15] In our study, untreated hepatitis improved significantly faster than treated hepatitis (23.2 *vs.* 63.8 days; p = 0.007). This faster improvement could thus explain the non-implementation of treatment for some patients. In addition, owing to the COVID pandemic and resulting non-COVID, unscheduled admissions, many patients improved before liver biopsy and without any treatment, but this was only after ICI discontinuation.

Consensus guidelines recommend treatment of 1–2 mg/kg/day methylprednisolone in high-grade hepatitis.[13,14] Recently, Li *et al.*[32] showed that treatment with 1 mg/kg/day methylprednisolone in high-grade CHILI provides similar hepatitis outcomes and reduces the risk of steroid-related complications in comparison with higher-dose regimens. Here our results support these findings and favour lower and delayed steroid therapy to allow spontaneous resolution in some cases and therefore reduce steroid-related adverse events.[32–34]

Seven patients were treated with UDCA alone in our cohort, and these patients were mainly with the cholestatic or mixed pattern. The indication of UDCA for treatment of CHILI is not defined, and recommendations are only based on severity according to CTCAE and do not consider the hepatitis pattern.[13,14] It seems essential to clarify the use of UDCA and potentially suggest it as first-line treatment in patients with the cholestatic pattern.[12,17] Because cholestatic CHILI shares similar histological features with primary biliary cholangitis and primary sclerosing cholangitis,[35] for which recommended treatment is with UDCA and steroids are not recommended, the idea of treating cholestatic CHILI with UDCA remains consistent.[36,37]

We report 15.3% of patients (n = 18) requiring second-line treatment. MMF was administered mainly in second-line treatment with a mean delay of 14.1 weeks for hepatitis resolution. MMF is a purine antagonist that inhibits the proliferation of lymphocytes infiltrating the liver and is considered as a first-line treatment option for corticosteroid-resistant CHILI in clinical guidelines (without randomised trial evidence).[38] Different second-line treatments have been reported (azathioprine, tacrolimus, ciclosporin, infliximab, and rituximab), but there is no consensus. Only a few cases of steroid-resistant hepatitis requiring second-line immunosuppression have been reported in the literature.[38]

Our study highlights the possibility of ICI rechallenge. We report ICI rechallenge in 43.6% of patients, and this was mostly with anti PD(L)-1 monotherapy (96.1%). Incriminated treatment rechallenge is generally contraindicated in DILI given the recurrence risk of severe forms.[23] With ICI, there is a potential risk of severe hepatitis in case of recurrence; however, in the oncological context, the benefit is often in favour of resuming ICI. Here, recurrence after rechallenge did not appear to be systematic; 23.5% of patients developed recurrent CHILI with ranging severity. Moreover, ICI rechallenge was even possible after severe hepatitis (CTCAE grade 3–4) among 31.6% of patients without an increased rate of CHILI recurrence. International guidelines recommend permanent ICI discontinuation for CTCAE grade ≥3 hepatitis.[14] However, many of these patients could in fact benefit from ICI maintenance. These data are consistent with previous studies reporting between 25.8 and 35% of hepatitis recurrence after ICI rechallenge.[19,39] In our study, only one patient (2%) presented a different and non-hepatic irAE after ICI rechallenge; this is inconsistent with literature reporting up to 65.2% of different irAEs after ICI rechallenge.[19] This difference could be explained by the collection of data on irAEs after ICI rechallenge in our study, which was only collected in patients with CHILI recurrence and perhaps during a shorter follow-up time. In our study, treatment with steroids or UDCA was still ongoing at the time of rechallenge in 68.6% of patients. Finally, neither the type of previous CHILI treatment nor the continuation of CHILI treatment after rechallenge affected the likelihood of CHILI recurrence.

We acknowledge some limitations to our study. Firstly, this is a retrospective study with the known biases related to this type of study. Secondly, the pathologists were different among the three different centres with no centralised tissue slide reading. To limit this bias, liver biopsy interpretation guidelines were validated by pathologists especially for this study (Table S7). Moreover, none of the patients included had a history of autoimmune disease before initiation of immunotherapy and there were no cases of liver metastasis after inclusion – two factors that would have increased the risk of recurrence. Finally, there is likely an over-representation of CTCAE grade ≥3 CHILI compared with grades 1 and 2 given that patient recruitment was based on discussions requiring multidisciplinary meetings.

Despite these limitations inherent to the retrospective design of our study, this work highlights the importance of separating CHILI into two different but equally frequent patterns (*i.e.* cholestatic and hepatocellular) with different outcomes. Characterisation of the hepatitis pattern could also guide the treatment of CHILI: steroids ± UDCA or UDCA alone. Further randomised studies should prospectively validate these findings and clarify the place of UDCA in cholestatic CHILI. Moreover, the possibility of a spontaneous favourable evolution in approximately 15% of patients and the feasibility of ICI rechallenge even after CTCAE grade ≥3 hepatitis are of particular value. Some patients with high-grade hepatitis may only have a favourable course by discontinuing ICI; immune-like inflammation is often transient without triggering a genuine autoimmune disease, in contradiction to what has been demonstrated by arthromuscular irAEs.[40] Future studies should focus on identifying the predictive factors of spontaneous recovery.

In conclusion, we report the first large study showing the different clinical patterns of CHILI. The cholestatic pattern was as frequent as the hepatocellular pattern, and this should lead to the consideration of remodelling current CHILI treatment recommendations. We also confirm that ICI rechallenge can be successful without having yet identified the predictive factors for recurrence.

Abbreviations

ALP, alkaline phosphatase; ALT, alanine aminotransferase; ANA, antinuclear antibodies; ASMA, antismooth muscle antibodies; AST, aspartate aminotransferase; CHILI, checkpoint inhibitor-induced liver injury; CTCAE, Common Terminology Criteria for Adverse Events; CTLA-4, cytotoxic T-lymphocyte-associated protein-4; DILI, drug-induced liver injury;

DILIN, Drug-Induced Liver Injury Network; GGT, gamma-glutamyl transferase; ICI, immune checkpoint inhibitor; irAE, immune-related adverse event; LAG-3, lymphocyte-activation gene 3; MMF, mycophenolate mofetil; PD-1, programmed cell death-1; PDL-1, programmed cell death ligand-1; RUCAM, Roussel Uclaf Causality Assessment Method; UDCA, ursodeoxycholic acid; ULN, upper limit of normal.

Financial support

The authors did not receive any funding for this research from agencies in the public, private, or non-profit sectors.

Conflicts of interest

The authors declare no conflicts of interest related to this work.

Please refer to the accompanying ICMJE disclosure forms for further details.

Authors' contributions

Study conception and design: LM, LH, CF, TC, ATJM. Data acquisition: LH, CF.

Data analysis and interpretation: LM, LH, CF, TC, ATJM. Manuscript preparation and drafting: LM, LH, CF, TC, ATJM. Statistical data analysis: AZ, LH. Manuscript reviewing: all authors. Have approved the final manuscript submitted: all authors.

Data availability statement

Data are available on request.

Supplementary data

Supplementary data to this article can be found online at https://doi.org/10.1016/j.jhepr.2023.100719.

References

[1] Sharma P, Allison JP. Immune checkpoint targeting in cancer therapy: toward combination strategies with curative potential. Cell 2015;161:205–214.
[2] Farkona S, Diamandis EP, Blasutig IM. Cancer immunotherapy: the beginning of the end of cancer? BMC Med 2016;14:73.
[3] Hodi FS, O'Day SJ, McDermott DF, Weber RW, Sosman JA, Haanen JB, et al. Improved survival with ipilimumab in patients with metastatic melanoma. N Engl J Med 2010;363:711–723.
[4] Gandhi L, Rodríguez-Abreu D, Gadgeel S, Esteban E, Felip E, De Angelis F, et al. Pembrolizumab plus chemotherapy in metastatic non-small-cell lung cancer. N Engl J Med 2018;378:2078–2092.
[5] Finn RS, Qin S, Ikeda M, Galle PR, Ducreux M, Kim TY, et al. Atezolizumab plus Bevacizumab in unresectable hepatocellular carcinoma. N Engl J Med 2020;382:1894–1905.
[6] André T, Shiu KK, Kim TW, Jensen BV, Jensen LH, Punt C, et al. Pembrolizumab in microsatellite-instability-high advanced colorectal cancer. N Engl J Med 2020;383:2207–2218.
[7] Doki Y, Ajani JA, Kato K, Xu J, Wyrwicz L, Motoyama S, et al. Nivolumab combination therapy in advanced esophageal squamous-cell carcinoma. N Engl J Med 2022;386:449–462.
[8] La-Beck NM, Jean GW, Huynh C, Alzghari SK, Lowe DB. Immune checkpoint inhibitors: new insights and current place in cancer therapy. Pharmacotherapy 2015;35:963–976.
[9] Tawbi HA, Schadendorf D, Lipson EJ, Ascierto PA, Matamala L, Castillo Gutiérrez E, et al. Relatlimab and nivolumab versus nivolumab in untreated advanced melanoma. N Engl J Med 2022;386:24–34.
[10] Haanen JBAG, Carbonnel F, Robert C, Kerr KM, Peters S, Larkin J, et al. Management of toxicities from immunotherapy: ESMO Clinical Practice Guidelines for diagnosis, treatment and follow-up. Ann Oncol 2017;28(Suppl. 4):iv119–iv142.
[11] De Martin E, Michot JM, Rosmorduc O, Guettier C, Samuel D. Liver toxicity as a limiting factor to the increasing use of immune checkpoint inhibitors. JHEP Rep 2020;2:100170.
[12] De Martin E, Michot JM, Papouin B, Champiat S, Mateus C, Lambotte O, et al. Characterization of liver injury induced by cancer immunotherapy using immune checkpoint inhibitors. J Hepatol 2018;68:1181–1190.
[13] Peeraphatdit TB, Wang J, Odenwald MA, Hu S, Hart J, Charlton MR. Hepatotoxicity from immune checkpoint inhibitors: a systematic review and management recommendation. Hepatology 2020;72:315–329.
[14] Dougan M, Wang Y, Rubio-Tapia A, Lim JK. AGA clinical practice update on diagnosis and management of immune checkpoint inhibitor colitis and hepatitis: expert review. Gastroenterology 2021;160:1384–1393.
[15] Gauci ML, Baroudjian B, Zeboulon C, Pages C, Poté N, Roux O, et al. Immune-related hepatitis with immunotherapy: are corticosteroids always needed? J Hepatol 2018;69:548–550.
[16] Gelsomino F, Vitale G, D'Errico A, Bertuzzi C, Andreone P, Ardizzoni A. Nivolumab-induced cholangitic liver disease: a novel form of serious liver injury. Ann Oncol 2017;28:671–672.
[17] Onoyama T, Takeda Y, Yamashita T, Hamamoto W, Sakamoto Y, Koda H, et al. Programmed cell death-1 inhibitor-related sclerosing cholangitis: a systematic review. World J Gastroenterol 2020;26:353–365.
[18] Doherty GJ, Duckworth AM, Davies SE, Mells GF, Brais R, Harden SV, et al. Severe steroid-resistant anti-PD1 T-cell checkpoint inhibitor-induced hepatotoxicity driven by biliary injury. ESMO Open 2017;2:e000268.
[19] Riveiro-Barciela M, Barreira-Díaz A, Callejo-Pérez A, Muñoz-Couselo E, Díaz-Mejía N, Díaz-González Á, et al. Retreatment with checkpoint inhibitors after a severe immune-related hepatitis: results from a prospective multicenter study. Clin Gastroenterol Hepatol 2023;21:732–740.
[20] Fontana RJ, Watkins PB, Bonkovsky HL, Chalasani N, Davern T, Serrano J, et al. Drug-Induced Liver Injury Network (DILIN) prospective study: rationale, design and conduct. Drug Saf 2009;32:55–68.
[21] Danan G, Benichou C. Causality assessment of adverse reactions to drugs – I. A novel method based on the conclusions of international consensus meetings: application to drug-induced liver injuries. J Clin Epidemiol 1993;46:1323–1330.
[22] Benichou C, Danan G, Flahault A. Causality assessment of adverse reactions to drugs – II. An original model for validation of drug causality assessment methods: case reports with positive rechallenge. J Clin Epidemiol 1993;46:1331–1336.
[23] European Association for the Study of the Liver. EASL clinical practice guidelines: drug-induced liver injury. J Hepatol 2019;70:1222–1261.
[24] Takinami M, Ono A, Kawabata T, Mamesaya N, Kobayashi H, Omori S, et al. Comparison of clinical features between immune-related sclerosing cholangitis and hepatitis. Invest New Drugs 2021;39:1716–1723.
[25] Berry P, Kotha S, Zen Y, Papa S, El Menabawey T, Webster G, et al. Immune checkpoint inhibitor-related cholangiopathy: novel clinicopathological description of a multi-centre cohort. Liver Int 2023;43:147–154.
[26] Pi B, Wang J, Tong Y, Yang Q, Lv F, Yu Y. Immune-related cholangitis induced by immune checkpoint inhibitors: a systematic review of clinical features and management. Eur J Gastroenterol Hepatol 2021;33(Suppl. 1):e858–e867.
[27] Stein S, Henze L, Poch T, Carambia A, Krech T, Preti M, et al. IL-17A/F enable cholangiocytes to restrict T cell-driven experimental cholangitis by upregulating PD-L1 expression. J Hepatol 2021;74:919–930.
[28] Zhang AL, Wang F, Chang LS, McDonnell ME, Min L. Coexistence of immune checkpoint inhibitor-induced autoimmune diabetes and pancreatitis. Front Endocrinol 2021;12:620522.
[29] Terashima T, Iwami E, Shimada T, Kuroda A, Matsuzaki T, Nakajima T, et al. IgG4-related pleural disease in a patient with pulmonary adenocarcinoma under durvalumab treatment: a case report. BMC Pulm Med 2020;20:104.
[30] Agrawal R, Guzman G, Karimi S, Giulianotti PC, Lora AJM, Jain S, et al. Immunoglobulin G4 associated autoimmune cholangitis and pancreatitis following the administration of nivolumab: a case report. World J Clin Cases 2022;10:7124–7129.
[31] Cohen JV, Dougan M, Zubiri L, Reynolds KL, Sullivan RJ, Misdraji J. Immune checkpoint inhibitor related liver injury: histopathologic pattern does not correlate with response to immune suppression. Mod Pathol 2021;34:426–437.
[32] Li M, Wong D, Vogel AS, Sack JS, Rahma OE, Hodi FS, et al. Effect of corticosteroid dosing on outcomes in high-grade immune checkpoint inhibitor hepatitis. Hepatol 2022;75:531–540.
[33] Faje AT, Lawrence D, Flaherty K, Freedman C, Fadden R, Rubin K, et al. High-dose glucocorticoids for the treatment of ipilimumab-induced hypophysitis is associated with reduced survival in patients with melanoma. Cancer 2018;124:3706–3714.

[34] Arbour KC, Mezquita L, Long N, Rizvi H, Auclin E, Ni A, et al. Impact of baseline steroids on efficacy of programmed cell death-1 and programmed death-ligand 1 blockade in patients with non-small-cell lung cancer. J Clin Oncol 2018;36:2872–2878.
[35] Visentin M, Lenggenhager D, Gai Z, Kullak-Ublick GA. Drug-induced bile duct injury. Biochim Biophys Acta Mol Basis Dis 2018;1864:1498–1506.
[36] European Association for the Study of the Liver. EASL Clinical Practice Guidelines: the diagnosis and management of patients with primary biliary cholangitis. J Hepatol 2017;67:145–172.
[37] European Association for the Study of the Liver. EASL clinical practice guidelines: management of cholestatic liver diseases. J Hepatol 2009;51:237–267.
[38] Ziogas DC, Gkoufa A, Cholongitas E, Diamantopoulos P, Anastasopoulou A, Ascierto PA, et al. When steroids are not enough in immune-related hepatitis: current clinical challenges discussed on the basis of a case report. J Immunother Cancer 2020;8:e001322.
[39] Patrinely Jr JR, McGuigan B, Chandra S, Fenton SE, Chowdhary A, Kennedy LB, et al. A multicenter characterization of hepatitis associated with immune checkpoint inhibitors. Oncoimmunology 2021;10:1875639.
[40] Kostine M, Finckh A, Bingham CO, Visser K, Leipe J, Schulze-Koops H, et al. EULAR points to consider for the diagnosis and management of rheumatic immune-related adverse events due to cancer immunotherapy with checkpoint inhibitors. Ann Rheum Dis 2021;80:36–48.

Journal of Hepatology, Volume *5*

Supplemental information

Clinical pattern of checkpoint inhibitor-induced liver injury in a multi-centre cohort

Lina Hountondji, Christophe Ferreira De Matos, Fanny Lebossé, Xavier Quantin, Candice Lesage, Pascale Palassin, Valérian Rivet, Stéphanie Faure, Georges-Philippe Pageaux, Éric Assenat, Laurent Alric, Amel Zahhaf, Dominique Larrey, Philine Witkowski Durand Viel, Benjamin Riviere, Selves Janick, Stéphane Dalle, Alexandre Thibault Jacques Maria, Thibaut Comont, and Lucy Meunier

Clinical pattern of checkpoint inhibitor-induced liver injury in a multicenter cohort

Lina Hountondji, Christophe Ferreira De Matos, Fanny Lebossé, Xavier Quantin, Candice Lesage, Pascale Palassin, Valérian Rivet, Stéphanie Faure, Georges-Philippe Pageaux, Éric Assenat, Laurent Alric, Amel Zahaf, Dominique Larrey, Philine Witkowski Durand Viel, Benjamin Riviere, Selves Janick, Stéphane Dalle, Alexandre Thibault Jacques Maria, Thibaut Comont[#], Lucy Meunier[#]

Table of contents:

Table S1: Clinico-biological comparison between cholestatic and hepatocellular patterns

Table S2: Clinico-biological features between immune-related sclerosing cholangitis and CHILI without macroscopic bile duct injury

Table S3: Comparison between treated and non-treated CHILI

Table S4: Comparison between steroids including treatment and other conditions

Table S5: Comparison between biopsied and non-biopsied patients

Table S6: Comparison between patients with and without rechallenge

Table S1: Clinico-biological comparison between cholestatic and hepatocellular patterns

	Cholestatic hepatitis n= 43 (36.8 %)	Hepatocellular hepatitis n= 45 (38.5 %)	*p value*
**Age (years), mean (SD)**	68	60	**0.004**
**Sex, n (%)**			0.133
Female	17 (19.3)	25 (28.4)	
Male	26 (29.5)	20 (22.7)	
**Cancer, n (%)**			0.091
Lung	15 (17)	10 (11.4)	
Melanoma	14 (15.9)	23 (26.1)	
Renal and urothelial	7 (8)	10 (11.4)	
Other cancer	7 (8)	2 (2.3)	
**Checkpoint inhibitor, n (%)**			**< 0.001**
Anti-PD1/PDL1	35 (39.8)	16 (18.2)	
Anti-CTLA4/Anti-LAG3	1 (1.1)	2 (2.3)	
Combotherapy with anti-CTLA4	7 (8)	27 (30.7)	
**Cycles of ICI infusion, mean (SD)**	4.9 (4.4)	3.5 (2.4)	0.64
**Time until onset (days), mean (SD)**	182.4 (262.5)	191.6 (372.1)	0.894
**RUCAM**			0.111
Possible (3-5)	0	4 (4.5)	
Probable (6-8)	28 (31.8)	24 (27.3)	
Highly probable (≥ 9)	15 (17)	17 (19.3)	
**Laboratory liver tests, mean/xULN (SD)**			
ALT	193.8 (151.9)	792.3 (1048.3)	**<0.001**
AST	166.4 (154.9)	535.3 (906.9)	**0.01**
GGT	670.7 (532.3)	202.1 (176.4)	**< 0.001**
ALP	804.4 (1687.2)	177.6 (124.9)	**0.015**
Total bilirubin	32.4 (45.7)	19.8 (24.2)	0.109
Jaundice (total bilirubin > N)	12 (10.3)	10 (11.4)	0.538
**Autoantibodies**			
ANA only	7 (8)	5 (5.7)	0.480
ASMA	0	5 (5.7)	**0.023**
**Bile duct injury, n (%)**	8 (9.1)	0	**0.002**
**Liver biopsy, n (%)**	20 (22.7)	23 (26.1)	0.666
**Histology, n (%)**			
Biliary injury	16 (39)	6 (14.6)	**< 0.001**
Granuloma	4 (10.3)	4 (10.3)	0.997
Endothelitis	0	3 (10.3)	**0.042**
Fibrin ring granuloma	1 (2.6)	0	0.299
**Other irAEs, n (%)**			
Extra hepatic irAE	20 (22.7)	13 (14.8)	0.088
Gastrointestinal	6 (6.8)	4 (4.5)	0.454
Cutaneous	4 (4.5)	7 (8)	0.375
Endocrine	4 (4.5)	3 (3.4)	0.648
Other irAE*	10 (11.4)	3 (3.4)	**0.028**
Multiple irAEs	7 (8)	6 (6.8)	0.171
**Hepatitis treatment, n (%)**			
Steroids only	15 (17)	31 (35.2)	**0.005**
UDCA only	5 (5.7)	0	**0.005**
Steroids + UDCA	18 (20.5)	10 (11.4)	**0.005**
Steroid including regimen	33 (37.5)	41 (46.6)	0.065
No treatment	5 (5.7)	4 (4.5)	0.672
Second line treatment	7 (8)	9 (10.2)	0.651
**Days until resolution to grade 1, mean (SD)**	69.5 (50)	59 (49.4)	0.488

Table S2: Clinico-biological features between immune-related sclerosing cholangitis and CHILI without macroscopic bile duct injury

	Bile duct stenosis n= 8 (6.8 %)	No bile duct stenosis 109 (93.2 %)	*p value*
**Age (years), mean (SD)**	65.3 (8.9)	63.2 (14.2)	0.686
**Sex, n (%)**			0.197
Female	2 (1.7)	52 (42.5)	
Male	6 (5.2)	55 (47.8)	
**Cancer, n (%)**			0.273
Lung	3 (2.6)	30 (26.1)	
Melanoma	1 (0.9)	47 (40.9)	
Renal and urothelial	2 (1.7)	20 (17.4)	
Other cancer	2 (1.7)	10 (8.7)	
**Checkpoint inhibitor, n (%)**			0.254
Anti-PD1/PDL1	7 (6.1)	62 (53.9)	
Anti-CTLA4/Anti-LAG3	0	4 (3.5)	
Combotherapy with anti-CTLA4	1 (0.9)	41 (35.7)	
**Cycles of ICI infusion, mean (SD)**	6.9 (3.4)	4.4 (4.6)	0.137
**Time until onset (days), mean (SD)**	236 (197.7)	173.6 (296.4)	0.560
**RUCAM**			0.754
Possible (3-5)	0	7 (6.1)	
Probable (6-8)	5 (4.3)	64 (55.7)	
Highly probable (≥ 9)	3 (2.6)	36 (31.3)	
**Laboratory liver tests, mean/xULN (SD)**			
ALT	180.5 (107.8)	463.1 (741.8)	0.286
AST	127.6 (136.6)	327.2 (620.2)	0.367
GGT	1027.4 (821.7)	353.3 (330.5)	**< 0.001**
ALP	1818.9 (3776.8)	319.7 (369.2)	**< 0.001**
Total bilirubin	18.2 (23.9)	24.2 (34.4)	0.628
Jaundice (total bilirubin > N)	2 (1.7)	26 (22.6)	0.964
**Autoantibodies**			
ANA only	0	16 (13.9)	0.238
ASMA	0	6 (5.3)	0.489
**Liver biopsy, n (%)**	6 (5.2)	43 (37.4)	0.055
**Histology, n (%)**			
Biliary injury	5 (10.6)	21 (44.7)	0.139
Granuloma	0	9 (20.4)	0.409
Endothelitis	0	4 (11.4)	0.334
Fibrin ring granuloma	0	1 (2.3)	0.688
**Other irAEs, n (%)**			
Extra hepatic irAE	5 (4.3)	42 (36.5)	0.197
Gastrointestinal	2 (1.7)	10 (8.7)	0.162
Cutaneous	0	13 (11.3)	0.295
Endocrine	2 (1.7)	9 (7.8)	0.124
Other irAE*	2 (1.7)	17 (14.8)	0.503
Multiple irAEs	1 (0.9)	19 (16.5)	**0.002**
**Hepatitis treatment, n (%)**			
Steroids only	1 (0.9)	60 (52.2)	**0.008**
UDCA only	1 (0.9)	6 (5.2)	**0.008**
Steroids + UDCA	6 (5.2)	25 (21.7)	**0.008**
Steroid including regimen	7 (6.1)	85 (73.9)	0.582
No treatment	0	16 (13.9)	0.238
Second line treatment	3 (2.6)	15 (13)	0.078
**Days until resolution to grade 1, mean (SD)**	124 (26.4)	50.5 (45)	**< 0.001**

Table S3: Comparison between treated and non-treated CHILI

	Treatment n= 100 (85.5 %)	No treatment n= 17 (14.5 %)	*p value*
**Age (years), mean (SD)**	63.6 (13.9)	61.2 (13.8)	0.512
**Sex, n (%)**			0.544
Female	45 (38.5)	9 (7.7)	
Male	55 (47)	8 (6.8)	
**Cancer, n (%)**			0.565
Lung	29 (24.8)	5 (4.3)	
Melanoma	44 (37.6)	5 (4.3)	
Renal and urothelial	18 (15.4)	4 (3.4)	
Other cancer	9 (7.7)	3 (2.6)	
**Checkpoint inhibitor, n (%)**			0.508
Anti-PD1/PDL1	58 (49.6)	12 (10.3)	
Anti-CTLA4/Anti-LAG3	4 (3.4)	0	
Combotherapy with anti-CTLA4	38 (32.5)	5 (4.3)	
**Pattern, n (%)**			0.066
Cholestatic	38 (32.5)	5 (4.3)	
Mixed	21 (17.9)	8 (6.8)	
Cytolytic	41 (35)	4 (3.4)	
**Cycles of ICI infusion, mean (SD)**	4.4 (4.7)	5.4 (4)	0.405
**Time until onset (days), mean (SD)**	181.1 (307)	144.6 (135.7)	0.632
**RUCAM**			0.456
Possible (3-5)	7 (6)	0	
Probable (6-8)	61 (52.1)	10 (8.5)	
Highly probable (≥ 9)	32 (27.4)	7 (6)	
**Laboratory liver tests, mean/xULN (SD)**			
ALT	469.4 (763.9)	284.4 (220.6)	0.325
AST	332.2 (640.5)	203.1 (161.6)	0.412
GGT	419.4 (435.1)	336.2 (288.4)	0.450
ALP	454.7 (1145.4)	245.5 (179.3)	0.455
Total bilirubin	25 (35.5)	15.6 (14.2)	0.299
Jaundice (total bilirubin > N)	26 (22.2)	2 (1.7)	0.203
**Autoantibodies**			
ANA only	14 (12)	2 (1.7)	0.804
ASMA	6 (5.2)	0	0.295
**Bile duct injury, n (%)**	8 (7)	0	0.238
**Liver biopsy, n (%)**	45 (38.5)	4 (3.4)	0.097
**Histology, n (%)**			
Biliary injury	23 (48.9)	3 (6.4)	0.408
Granuloma	8 (18.1)	1 (2.3)	0.148
Endothelitis	4 (11.4)	0	0.515
Fibrin ring granuloma	1 (2.3)	0	0.784
**Other irAEs, n (%)**			
Extra hepatic irAE	41 (35)	7 (6)	0.989
Gastrointestinal	12 (10.3)	0	0.132
Cutaneous	13 (11.1)	0	0.115
Endocrine	10 (8.5)	1 (0.9)	0.591
Other irAE*	18 (15.4)	2 (1.7)	0.528
Multiple irAEs	19 (16.2)	1 (0.9)	0.121
**Days until resolution to grade 1, mean (SD)**	63.8 (49.9)	23.2 (14.6)	**0.007**
**Cancer status (RECIST 1.1), n (%)**			0.278
Progressive disease	23 (20)	7 (6.1)	
Stable disease	13 (11.3)	4 (3.5)	
Partial response	34 (29.6)	4 (3.5)	
Complete response	20 (17.4)	1 (0.9)	
**CHILI recurrence, n (%)**	9 (19.6)	3 (6.5)	0.153

Table S4: Comparison between steroids including treatment and other conditions*

	Steroids including regimen n= 93 (79.5 %)	Other treatments n= 24 (20.5 %)	*p value*
**Age (years), mean (SD)**	63.4 (14.1)	62.7 (13)	0.827
**Sex, n (%)**			0.672
Female	42 (35.9)	12 (10.3)	
Male	51 (43.6)	12 (10.3)	
**Cancer, n (%)**			0.193
Lung	26 (22.2)	8 (6.8)	
Melanoma	42 (35.9)	7 (6)	
Renal and urothelial	18 (15.4)	4 (3.4)	
Other cancer	7 (6)	5 (4.3)	
**Checkpoint inhibitor, n (%)**			0.082
Anti-PD1/PDL1	51 (43.6)	19 (16.2)	
Anti-CTLA4/Anti-LAG3	4 (3.4)	0	
Combotherapy with anti-CTLA4	38 (32.5)	5 (4.3)	
**Pattern, n (%)**			**0.025**
Cholestatic	33 (28.2)	10 (8.5)	
Mixed	19 (16.2)	10 (8.5)	
Hepatocellular	41 (35)	4 (3.4)	
**Cycles of ICI infusion, mean (SD)**	4.5 (4.8)	4.7 (3.6)	0.799
**Time until onset (days), mean (SD)**	169.7 (294.9)	199.2 (266.4)	0.658
**RUCAM**			0.292
Possible (3-5)	7 (6)	0	
Probable (6-8)	57 (48.7)	14 (12)	
Highly probable (≥ 9)	29 (24.8)	10 (8.5)	
**Laboratory liver tests, mean/xULN (SD)**			
ALT	488.6 (787.7)	263.9 (208.9)	0.170
AST	339.1 (661.1)	213.9 (182.6)	0.361
GGT	393.9 (410.8)	458.9 (444.2)	0.506
ALP	441.4 (1167.9)	356 (484.8)	0.726
Total bilirubin	24.9 (36.1)	18.7 (19.6)	0.429
Jaundice (total bilirubin > N)	24 (20.5)	4 (3.4)	0.349
**Autoantibodies**			
ANA only	13 (11.1)	3 (2.6)	0.851
ASMA	6 (5.2)	0	0.196
**Bile duct injury, n (%)**	7 (6.1)	1 (0.9)	0.582
**Liver biopsy, n (%)**	42 (35.9)	7 (6)	0.157
**Histology, n (%)**			
Biliary injury	21 (44.7)	5 (10.6)	0.353
Granuloma	8 (18.1)	1 (2.3)	0.378
Endothelitis	4 (11.4)	0	0.334
Fibrin ring granuloma	1 (2.3)	0	0.688
**Other irAEs, n (%)**			
Extra hepatic irAE	38 (32.5)	10 (8.5)	0.943
Gastrointestinal	12 (10.3)	0	0.063
Cutaneous	13 (11.1)	0	0.052
Endocrine	9 (7.7)	2 (1.7)	0.841
Other irAE*	16 (13.7)	4 (3.4)	0.950
Multiple irAEs	19 (16.2)	1 (0.9)	0.080
**Second-line immunosuppressant, n (%)**	18 (15.4)	0	**0.019**
**Days until resolution to grade 1, mean (SD)**	23.2 (14.6)	63.8 (49.9)	**0.007**
**Cancer status (RECIST 1.1), n (%)**			0.222
Progressive disease	21 (18.3)	9 (7.8)	
Stable disease	12 (10.4)	5 (4.3)	
Partial response	31 (27)	7 (6.1)	
Complete response	20 (17.4)	1 (0.9)	

**CHILI recurrence, n (%)**	8 (17.4)	4 (8.7)	0.257

*UDCA only or no treatment

Table S5: Comparison between biopsied and non-biopsied patients

	Biopsy n= 49 (41.9 %)	No biopsy n= 68 (58.1 %)	*p value*
**Age (years), mean (SD)**	62.8 (15)	63.6 (13)	0.758
**Sex, n (%)**			0.370
Female	25 (21.4)	29 (24.8)	
Male	24 (20.5	39 (33.3)	
**Cancer, n (%)**			0.109
Lung	13 (11.1)	21 (17.9)	
Melanoma	19 (16.2)	30 (25.6)	
Renal and urothelial	14 (12)	8 (6.8)	
Other cancer	3 (2.6)	9 (7.7)	
**Checkpoint inhibitor, n (%)**			0.243
Anti-PD1/PDL1	26 (22.2)	44 (37.6)	
Anti-CTLA4/Anti-LAG3	3 (2.6)	1 (0.9)	
Combotherapy with anti-CTLA4	20 (17.1)	23 (19.7)	
**Cycles of ICI infusion, mean (SD)**	4.4 (4.1)	4.5 (4.9)	0.892
**Time until onset (days), mean (SD)**	184.8 (233.6)	169.2 (323.7)	0.774
**RUCAM**			0.427
Possible (3-5)	2 (1.7)	5 (4.3)	
Probable (6-8)	33 (28.2)	38 (32.5)	
Highly probable (≥ 9)	14 (12)	25 (21.4)	
**Laboratory liver tests, mean (SD)**			
ALT	608.1 (1010.2)	323.2 (340)	**0.033**
AST	415.02 (857.3)	240.2 (276.7)	0.118
GGT	526.2 (516.1)	317.1 (295.7)	0.007
ALP	577.2 (1572.3)	314.2 (393.9)	0.188
Total bilirubin	31.6 (45)	17.8 (19.6)	**0.028**
Jaundice (total bilirubin > N)	16 (13.7)	12 (10.3)	0.061
**Autoantibodies**			
ANA only	7 (6)	9 (7.7)	0.870
ASMA	4 (3.5)	2 (1.7)	0.221
**Bile duct injury, n (%)**	6 (5.2)	2 (1.7)	0.055
**Other irAEs, n (%)**			
Extra hepatic irAE	15 (12.8)	33 (28.2)	0.052
Gastrointestinal	3 (2.6)	9 (7.7)	0.211
Cutaneous	4 (3.4)	9 (7.7)	0.389
Endocrine	4 (3.4)	7 (6)	0.697
Other irAE	5 (4.3)	15 (12.8)	0.093
Multiple irAEs	6 (5.1)	14 (12)	0.244
**Hepatitis treatment, n (%)**			
Corticoids only	3 (18.8)	40 (34.2)	0.19
UDCA only	3 (2.6)	4 (3.4)	0.19
Corticoids + UDCA	20 (17.1)	11 (9.4)	0.19
Corticoids including regimen	42 (35.9)	51 (43.6)	0.157
No treatment	4 (3.4)	13 (11.1)	0.097
Second line treatment	16 (13.7)	2 (1.7)	**< 0.001**
**Days until resolution to grade 1, mean (SD)**	75.2 (54.5)	42.4 (37.7)	0.006

Table S6: Comparison between patients with and without rechallenge

	Rechallenge n= 51 (43.6%)	No rechallenge n= 66 (56.4%)	*p value*
**Age (years), mean (SD)**	61.3 (15.3)	64.7 (12.5)	0.182
**Sex, n (%)**			0.863
Female	24 (20.5)	30 (25.6)	
Male	27 (23.1)	36 (30.8)	
**Pre-existing liver disease, n (%)**			
Cirrhosis	1 (0.9)	4 (3.4)	0.277
**Cancer, n (%)**			0.644
Lung	15 (12.8)	19 (16.2)	
Melanoma	23 (19.7)	26 (22.2)	
Renal and urothelial	7 (6)	15 (12.8)	
Other cancer	6 (5.1)	6 (5.1)	
**Checkpoint inhibitor, n (%)**			0.197
Anti-PD-1/PDL-1	31 (26.5)	39 (33.3)	
Anti-CTLA-4/Anti-LAG-3	0	4 (3.4)	
Combi-therapy with anti-CTLA-4	20 (17.1)	23 (19.7)	
**Cycles of ICI infusion, mean (SD)**	4.4 (5)	4.6 (4.3)	0.894
**Time until onset (days), mean (SD)**	137.5 (198.2)	205.4 (340.8)	0.208
**Autoantibodies**			
ANA only	9 (7.7)	7 (6)	0.272
ASMA	2 (1.7)	4 (3.5)	0.577
**Pattern, n (%)**			0.312
Cholestatic	16 (13.7)	27 (23.1)	
Mixed	16 (13.7)	13 (11.1)	
Hepatocellular	19 (16.2)	26 (22.2)	
**Severity (CTCAE), n (%)**			0.006
Grade 1	1 (0.9)	3 (2.6)	
Grade 2	13 (11.1)	4 (3.4)	
Grade 3	32 (27.4)	41 (35)	
Grade 4	5 (4.3)	18 (15.4)	
**Jaundice (total bilirubin > N)**	9 (7.7)	19 (16.2)	0.161
**Bile duct injury, n (%)**	3 (2.6)	5 (4.3)	0.686
**Liver biopsy, n (%)**	13 (11.1)	36 (30.8)	0.002
**Histology, n (%)**			
Biliary injury	6 (12.8)	20 (42.6)	0.435
Granuloma	5 (11.4)	4 (9)	0.093
Endothelitis	1 (2.9)	3 (8.6)	0.593
Fibrin ring granuloma	1 (2.3)	0	0.118
**Other irAEs, n (%)**			
Extra hepatic irAE	19 (16.2)	29 (24.8)	0.466
Gastrointestinal	4 (3.4)	8 (6.8)	0.449
Cutaneous	8 (6.8)	5 (4.3)	0.166
Endocrine	6 (5.1)	5 (4.3)	0.441
Other irAE*	7 (6)	13 (11.1)	0.395
Multiple irAEs (>2)	10 (8.5)	10 (8.5)	0.397
**Hepatitis treatment, n (%)**			
Corticoids including regimen	39 (33.3)	54 (46.2)	0.477
No treatment	8 (6.8)	9 (7.7)	0.755
Other immunosuppressant	5 (4.3)	13 (11.1)	0.141
**Days until resolution to grade 1, mean (SD)**	47.5 (36.9)	65.5 (56.7)	0.136
**Cancer status (RECIST 1.1), n (%)**			0.533
Progressive disease	14 (12.2)	16 (13.9)	
Stable disease	9 (7.8)	8 (7)	
Partial response	17 (14.8)	21 (18.3)	
Complete response	6 (5.2)	15 (13)	
Unmesurable	5 (4.3)	4 (3.5)	

Table S7 : Histology

	Biopsied patients n= 49 (%)
**Inflammation and necrosis**	
Portal inflammation	35 (71.4)
Portal necrosis	9 (18.4)
Lobular inflammation	41 (83.7)
Lobular necrosis	24 (49)
Centrilobular injury	14 (28.6)
Panlobular injury	17 (34.7)
Interface hepatitis	5 (10.2)
Portal endothelitis	3 (6.1)
Centro venular endothelitis	1 (2)
**Cellular infiltrate**	46 (93.9)
Lymphocytes	34 (69.4)
Neutrophils	21 (42.9)
Eosinophils	25 (51)
Histiocytes	16 (32.7)
Macrophages	20 (40.8)
Plama cells	13 (26.5)
Granuloma	10 (20.4)
Fibrin ring granuloma	1 (2)
Biliary injury	26 (53)
Bilirubinostasis	4 (8.1)
Ductopenia	2 (4.1)
Ductular reaction	10 (20.4)
Ductular dystrophia	13 (26.5)
Inflammatory cholangitis	15 (30.6)
Lymphocytic cholangitis	9 (18.4)
Neutrophilic cholangitis	2 (4.1)
Non-suppurative destructive cholangitis	3 (6.1)
Granulomatous cholangitis	2 (4.1)
Obstructive fibrous pericholangiolitis	0
**Fibrosis**	16 (32.7)
F0	31 (63.3)
Portal fibrosis (F1)	11 (22.4)
Centrolobular fibrosis (F1)	5 (10.2)
Fibrosis with septa (F2)	0
Fibrosis with numerous septa (F3)	0
Cirrhosis (F4)	1 (2)
